# Extracellular Vesicle Protein and MiRNA Signatures as Biomarkers for Post-Infectious ME/CFS Patients

**DOI:** 10.3390/ijms27052314

**Published:** 2026-02-28

**Authors:** Martina Seifert, Johannes Schäfers, Fiona F. Douglas, Carl Schwarzburg, Diana Boristowski, Anne Birke, Oliver Klein, Franziska Sotzny, Kerstin Rubarth, Lara Windzio, Christien M. Beez, Claudia Kedor Peters, Kirsten Wittke, Carmen Scheibenbogen, Anna Greco

**Affiliations:** 1 Institute for Medical Immunology, Charité-Universitätsmedizin Berlin, Corporate Member of Freie Universität Berlin and Humboldt Universität zu Berlin, 10117 Berlin, Germany; johannes.schaefers@charite.de (J.S.); fiona.douglas@fu-berlin.de (F.F.D.); schwarzburg.carl@gmail.com (C.S.); diana.boristowski@gmail.com (D.B.); birke@vrc.uni-frankfurt.de (A.B.); franziska.sotzny@charite.de (F.S.); lara.windzio@charite.de (L.W.); claudia.kedor@charite.de (C.K.P.); kirsten.wittke@charite.de (K.W.); carmen.scheibenbogen@charite.de (C.S.); anna.greco@charite.de (A.G.); 2 BIH-Center for Regenerative Therapies (BCRT), Berlin Institute of Health (BIH) at Charité-Universitätsmedizin Berlin, 13353 Berlin, Germany; oliver.klein@bih-charite.de; 3 German Centre for Cardiovascular Research (DZHK), 10785 Berlin, Germany; 4 Institute of Biometry and Clinical Epidemiology, Charité-Universitätsmedizin Berlin, Corporate Member of Freie Universität Berlin and Humboldt Universität zu Berlin, 10117 Berlin, Germany; kerstin.rubarth@charite.de; 5 Department of Cardiothoracic and Vascular Surgery, Deutsches Herzzentrum der Charité (DHZC), 13353 Berlin, Germany; christien.beez@dhzc-charite.de

**Keywords:** extracellular vesicles, protein cargo, miRNA, post-COVID syndrome, Myalgic Encephalomyelitis/Chronic Fatigue Syndrome

## Abstract

Post-infectious Myalgic Encephalomyelitis/Chronic Fatigue Syndrome (ME/CFS) is a chronic disease with unresolved pathophysiology and limited diagnostic options. Extracellular vesicles (EVs) carry disease-specific protein and miRNA signatures and may enable improved disease profiling. We aimed to identify novel protein and miRNA markers as potential biomarkers in plasma EVs from female ME/CFS patients, including post-COVID-19 ME/CFS and post-infectious ME/CFS of other origins, compared with healthy controls. EVs were isolated from plasma by size-exclusion chromatography and characterized for number, size, morphology, and surface marker expression. Flow cytometry showed that small EVs strongly expressed tetraspanins, with only minor differences between ME/CFS patients and healthy donors. Proteomic profiling of EVs from ME/CFS patients identified altered cargo proteins, including hemoglobin subunit alpha and insulin-like growth factor-binding protein acid labile subunit compared with healthy controls (*n* ≤ 10/cohort). Small RNA sequencing followed by qPCR revealed significant downregulation of hsa-let-7b-5p in EVs from post-COVID-19 ME/CFS patients (*n* = 12) versus healthy controls (*n* = 15). Reduced hsa-let-7b-5p expression correlated with impaired physical functioning and increased fatigue, pain, and immune activation. These findings indicate that EV cargo differences, particularly hemoglobin subunit alpha and insulin-like growth factor-binding protein acid labile subunit, as well as hsa-let-7b-5p, represent promising candidates for ME/CFS diagnosis and patient stratification.

## 1. Introduction

Myalgic Encephalomyelitis/Chronic Fatigue Syndrome (ME/CFS) is a debilitating, chronic disease affecting multiple systems within the body. Patients suffer from a variety of symptoms, including profound fatigue, cognitive and autonomic impairment, pain, and post-exertional malaise (PEM), resulting in severe impairment of quality of life [1]. In its most severe form, ME/CFS leads to substantial limitations in daily functioning, including profound occupational disability and dependence on caregiver support [2]. Recent global estimates suggest a prevalence of up to 1%, with females accounting for approximately 75–85% of ME/CFS cases [3]. ME/CFS can be triggered by various infections, including Epstein–Barr virus, influenza virus, human herpes virus, and also SARS-CoV-2 [4,5,6]. The COVID-19 pandemic and the associated Post-COVID Syndrome (PCS), often referred to as Long-COVID, have brought increased attention to ME/CFS. PCS shares significant clinical similarities with ME/CFS, particularly in terms of persistent fatigue, cognitive impairment and PEM, highlighting the overlap and potential common underlying mechanisms between these conditions. However, only a subgroup of PCS patients meets the diagnostic criteria for ME/CFS [7,8]. The pathomechanism of the ME/CFS disease is not yet fully understood [9,10]. Dysregulation of the immune system, disturbances of the autonomic nervous system and vascular function, cognitive impairment, and metabolic disturbances contribute to this complex multisystemic syndrome [3,6]. The heterogeneity in symptom severity and comorbidities among patients, influenced by diverse disease triggers and individual responses, further exacerbates the complexity. Consequently, research efforts must consider the interplay of various biological systems rather than focusing on a single etiological factor [11,12,13,14]. Recent studies increasingly support the concept that overlapping disease pathologies linked to ME/CFS and PCS, as well as their combined effects, contribute to the observed heterogeneity of the condition [15]. Current research is directed at deciphering how these system-wide disruptions arise and at identifying common molecular pathways. Studies have demonstrated biomedical dysregulations across multiple organ systems, including metabolic, muscular, cardiovascular and gastrointestinal disturbances [16,17,18,19,20]. Abnormalities in mitochondrial function and energy production have been documented in ME/CFS and may contribute to the profound fatigue experienced by patients [11].

At present, ME/CFS diagnosis relies exclusively on internationally agreed clinical criteria, as no specific biomarkers are available [21,22]. Over the years, various attempts have been made to discover potential biomarkers for ME/CFS, so far with limited success [23]. In a variety of other diseases, including COVID-19, EVs have recently gained considerable interest as carriers of soluble mediators [24,25]. EVs represent a complex group of small, bilayered membranous vesicles produced by various cell types. Initially, EV classification was based on size and biogenesis, resulting in three main groups (exosomes, microvesicles, apoptotic bodies/EVs), with the smallest EVs ranging between 50 and 150 nm [22]. Despite the distinct features and subtypes of EVs, all share certain surface markers, namely the tetraspanins CD9, CD63, and CD81 [26]. EVs are present in almost all body fluids and reflect characteristics of their parent cells [27]. They play crucial roles in intercellular communication and are pivotal in various biological processes, including disease and infections, due to their involvement in both innate and adaptive immune response [28]. The cargo of EVs includes proteins, DNA, lipids, or RNA, such as messenger RNA or miRNA [27]. MiRNAs have garnered particular interest owing to their influence on a wide range of infection-triggered diseases and relative gene expression regulation processes [29,30,31]. EVs can modulate vascular inflammation processes through their secretion, as well as through their cargo, which is altered during inflammatory states [32,33,34].

Studies investigating EVs in ME/CFS patients have revealed aberrant EV profiles compared to healthy controls (HCs). Specifically, several studies found significantly increased levels of circulating EVs in ME/CFS patients compared to HCs [35,36]. Additionally, other studies focused on the protein cargo of the plasma EVs. In 2019, Eguchi et al. identified a distinct EV protein profile in ME/CFS, enriched for proteins involved in focal adhesion, regulation of the actin cytoskeleton, the phosphoinositide 3-kinase (PI3K)–Akt signaling pathway, and EBV infection [37]. Moreover, the cytokine cargo of EVs was investigated, and differences in inter-cytokine correlations were found in the ME/CFS group compared to HCs [38]. In recent studies, the EV proteome of ME/CFS patients in comparison to HCs was analyzed before and after maximal physical exercise [39,40]. Herein, exercise elicited differential changes in the EV proteome of ME/CFS patients relative to HCs, with several altered EV proteins correlating with symptom severity. These findings suggest alterations in EV signaling dynamics involved in processes including blood coagulation and muscle contraction, energy metabolism, the complement system, and the stress response of the endoplasmic reticulum. Differentially expressed proteins found in this study could be associated with PEM in ME/CFS patients. As these studies were conducted using pre-pandemic cohorts, it remains to be determined whether similar EV alterations occur in patients who developed ME/CFS following COVID-19. Initial studies were performed to analyze the spectrum of miRNAs in serum or plasma of ME/CFS patients compared to HCs [41,42]. A computational analysis of published miRNA data from ME/CFS patients by Kaczmarek et al., which included findings from other studies [41,43], revealed that vascular endothelial growth factor A was the primary target of upregulated circulating miRNAs [43]. Another study identified a specific miRNA pattern associated with symptom severity in severely ill ME/CFS patients following PEM induction [44].

Despite encouraging results in the search for either specific protein or miRNA markers for ME/CFS in serum or plasma EVs, no unique marker for post-infectious ME/CFS has yet been identified or validated in independent cohorts. Therefore, in the present exploratory study, we aimed to identify and confirm differentially expressed protein and miRNA markers of plasma EVs from post-infectious ME/CFS patients. These markers were initially identified by us in exploratory study cohorts and subsequently verified in validation cohorts. In addition, we aimed to compare the potential markers in ME/CFS after COVID-19 with those following another disease trigger. Here, we provide both protein and miRNA candidate markers for post-infectious ME/CFS, as we believe they could be instrumental in improving patient diagnosis in future large-scale cohort studies.

## 2. Results

### 2.1. Study Design

**Figure 1:**
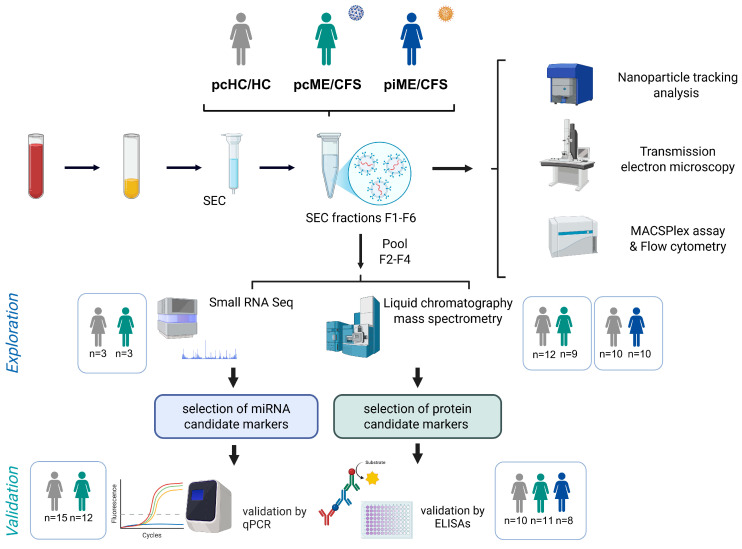
Schematic overview of the experimental study design. Acid-citrate-dextrose plasma from all study cohorts (pcHC/HC, pcME/CFS, piME/CFS) was used to isolate EVs by size-exclusion chromatography (SEC). EVs were characterized by nanoparticle tracking analysis, transmission electron microscopy, and flow cytometric analysis of surface marker expression using the MACSPlex assay. Pooled SEC fractions (F2–F4) from EV isolations were screened in exploratory cohorts by small RNA sequencing and liquid chromatography–mass spectrometry-based proteomics to identify both, differentially transported miRNAs and proteins. Selected candidate markers were tested in validation cohorts by qPCR and ELISA, respectively. Created with BioRender.com.

Plasma samples were collected from three study groups: post-COVID-19 ME/CFS (pcME/CFS) patients, post-infectious ME/CFS (piME/CFS) patients and the corresponding healthy controls with self-reported SARS-CoV2 infection (pcHC) and pre-pandemic healthy donors (HC), respectively (see Figure 1). EVs were isolated from all samples using size-exclusion chromatography (SEC). To ensure the quality and purity of the isolated EVs, SEC fractions were characterized by nanoparticle tracking analysis (NTA), transmission electron microscopy (TEM) and marker expression profiles (including tetraspanins) using the MACSPlex Exosome Kit and flow cytometric analysis. Pooled EV fractions from the exploratory cohorts were further examined by RNA sequencing (see Supplementary Table S1) and liquid-separation electrospray ionization mass spectrometry (liquid chromatography–mass spectrometry; see Supplementary Tables S2 and S3) to profile miRNA and protein cargo, respectively. Identified miRNA and protein markers of interest were subsequently analyzed in independent validation cohorts by enzyme-linked immunosorbent assay (ELISA) or qPCR. The female patient and control groups in the validation cohorts for protein markers (Table 1) and miRNA markers (Table 2) were similar in their age and body mass index. The piME/CFS group was younger and had a longer disease duration (median 24.45 months vs. 10 months in pcME/CFS). For the HCs, the self-reported time since the last SARS- CoV-2 infection was approximately 10 months. Both patients study groups showed similar disease and symptom severity (Table 1).

### 2.2. Isolated Plasma EVs from Both ME/CFS Patients and HCs Show Expected Characteristics

**Figure 2:**
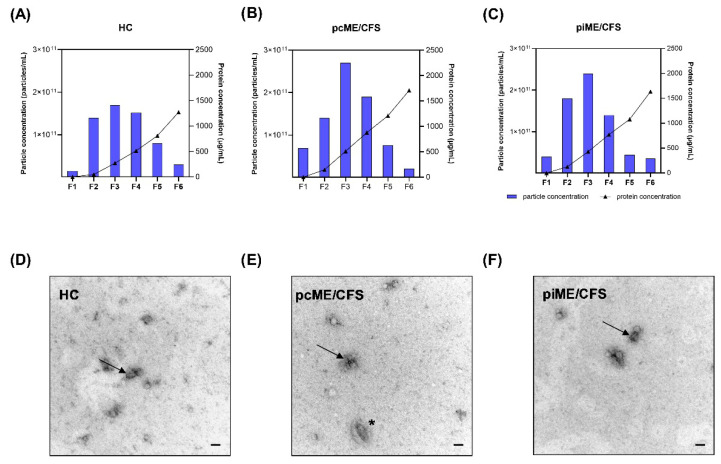
Characteristics of isolated plasma EVs from all cohorts. The particle concentration and protein concentration for each SEC fraction (F1–F6) were determined by NTA and BCA for representative samples of HC, pcME/CFS, and piME/CFS cohorts, respectively. Shown are the number of particles/mL (left *y*-axis) and protein concentration in μg/mL (right *y*-axis) for (**A**) HC, (**B**) pcME/CFS, and (**C**) piME/CFS. EVs from pooled SEC fractions F2–F4 of HCs, pcME/CFS, and piME/CFS patients were examined by TEM. Representative images are shown for (**D**) HC, (**E**) pcME/CFS, and (**F**) piME/CFS. In some cases, EVs clustered (black arrow) and showed a cup-shaped morphology (asterisk). Scale bars: 100 nm. HC = healthy control; pcME/CFS = post-COVID-19 ME/CFS; piME/CFS = post-infectious ME/CFS.

Acid-citrate-dextrose (ACD) plasma was collected and used for EV isolation by SEC on IZON qEV columns, as shown in the overview scheme (Figure 1). Particle concentrations and diameter size were analyzed by NTA, and protein content was determined by bicinchoninic acid. Shown are the NTA and BCA profiles for a representative HC donor (Figure 2A), a pcME/CFS patient (Figure 2B), and a piME/CFS patient (Figure 2C) for each fraction (F1-F6) of the SEC isolation. The predominant EV-containing fractions corresponded to F2–F4 in all study groups. As expected for SEC-based EV isolation, protein concentrations were minimal in F1 and gradually increased toward protein-rich late fractions (F5–F6), reaching approximately 1000–2000 mg/mL in F6. To optimize particle-to-protein ratios, fractions F2-F4 were pooled for all further analyses. The pooled fractions were used to verify the typical morphology and size of the isolated particles by transmission electron microscopy (TEM, Figure 2). Across all study groups, EVs displayed characteristic cup-shaped morphology and occasional vesicle clustering, as illustrated by representative TEM images for HC, pcME/CFS, and piME/CFS samples (Figure 2D–F).

**Figure 3:**
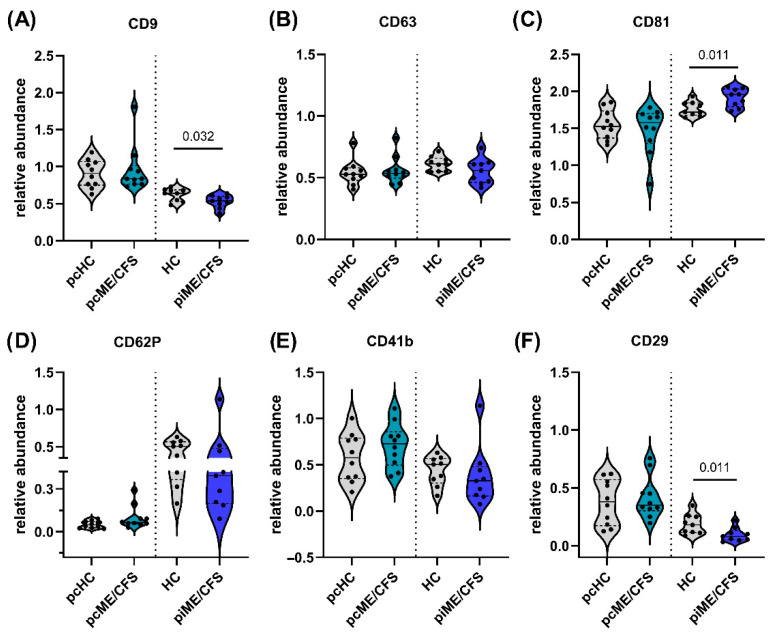
Relative abundance of extracellular vesicle (EV) surface markers in ME/CFS cohorts. EVs isolated from post-COVID-19 ME/CFS (pcME/CFS) and post-infectious ME/CFS (piME/CFS) patients and their respective healthy controls were analyzed using a bead-based multiplex flow cytometry assay. The relative abundance of each surface marker was calculated by normalizing the median fluorescence intensity (MFI) of each analyte to the combined MFI of the three tetraspanins CD9, CD63, and CD81, as described in the Methods. Shown are the medians with interquartile ranges and individual data points for (**A**) CD9, (**B**) CD63, (**C**) CD81, (**D**) CD62P, (**E**) CD41b, and (**F**) CD29. Comparisons between pcHC and pcME/CFS, and between pre-pandemic HC and piME/CFS, are separated by a vertical dotted line. Statistical significance was assessed using the Mann–Whitney U test, with *p*-values indicated. Sample sizes were pcHC (*n* = 10), pcME/CFS (*n* = 10), HC (*n* = 9), and piME/CFS (*n* = 9).

The presence of the three characteristic tetraspanin molecules (CD9, CD63, CD81) in isolated EVs of all study groups was verified using a bead-based flow cytometry assay. Indeed, CD9 (Figure 3A), CD63 (Figure 3B), and CD81 (Figure 3C) were observed at similar fluorescence intensities on the isolated EVs of each study group, with CD81 showing the highest relative abundance and CD63 the lowest. Notably, EVs from the piME/CFS patient group showed significantly (*p* = 0.011) higher levels of CD81 (Figure 3C) and significantly (*p* = 0.032) lower CD9 detection (Figure 3A) compared to corresponding HC-EVs. Together, the presence of these tetraspanins across all cohorts confirms successful EV isolation. Additionally, 37 other cellular markers were analyzed in parallel, as depicted exemplarily in Figure S1, displaying expression profiles for representative HC, pcME/CFS, and piME/CFS EV samples. Several markers were only detectable at very low levels or not at all in EV preparations, whereas others showed a clear presence in HC and patient groups (Figure 3D–F and Figure S1), with only marginal differences, as shown for selected markers CD62P (Figure 3D), CD41b (Figure 3E), and CD29 (Figure 3F). The endothelial marker CD62P tended to be more abundant (*p* = 0.089) in pcME/CFS than in HC-EVs (Figure 3D). Integrin CD29 was significantly (*p* = 0.011) lower expressed in piME/CFS-EVs compared to HC-EVs (Figure 3F).

### 2.3. Identification of Distinct Proteomic Signatures for pcME/CFS and piME/CFS in Exploratory Cohorts

Two independent mass spectrometry analyses in exploratory cohorts detected a total of 416 proteins in the comparison of pcME/CFS to pcHC and 278 proteins in the comparison of piME/CFS and pre-pandemic HCs. An initial Search Tool for the Retrieval of Interacting Genes/Proteins (STRING) database analysis was performed for all study groups separately based on gene ontology (GO) terms categorized into groups of proteins: cellular component, biological processes, subcellular localization and (patho)-physiology. GO-terms not meeting the defined criteria, as described in the Methods section, were excluded along with their associated proteins, resulting in a set of 192 and 180 proteins for the pcME/CFS and pcHC study groups, respectively. Out of the initially detected 278 proteins found in the piME/CFS and pre-pandemic HC dataset, 154 and 128 proteins for the respective study groups remained after filtering. Afterwards, unique protein candidates for all study groups were identified, as shown in Supplementary Table S4. One particular protein, the insulin-like growth factor acid labile subunit (ALS/IGFALS), was detected as a unique candidate protein in both ME/CFS patient groups.

**Figure 4:**
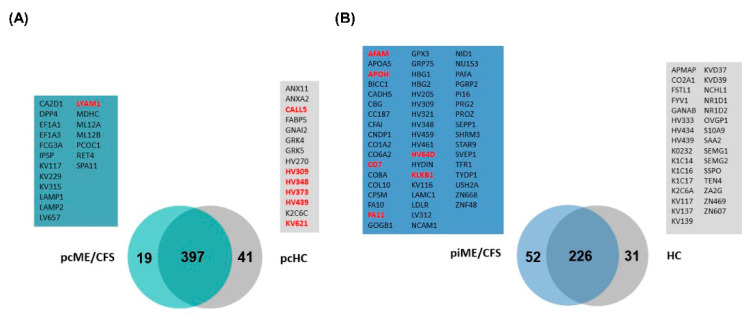
Unique EV protein candidates identified in ME/CFS cohorts. Venn diagrams show EV-associated proteins from (**A**) the post-COVID-19 ME/CFS (pcME/CFS) cohort compared with its matched post-COVID healthy controls (pcHC), and (**B**) the post-infectious ME/CFS (piME/CFS) cohort compared with pre-pandemic healthy controls (HC). Proteins uniquely detected in ≥3 individuals within each respective ME/CFS group are highlighted in red. Sample sizes were pcHC (*n* = 9), pcME/CFS (*n* = 12), HC (*n* = 10), and piME/CFS (*n* = 10).

In a second approach, all EV proteins initially found by mass spectrometry analysis were depicted as a VENN diagrams (Figure 4) to compare EV proteins between piME/CFS vs. HC (Figure 4A) and between pcME/CFS vs. pcHC (Figure 4B). As expected, a substantial proportion of EV proteins overlapped between the corresponding patient and control groups in each proteomic dataset. Moreover, several candidate proteins in the mass spectrometry analyses were consistent with those highlighted by the STRING-based qualitative assessment. Interestingly, ALS/IGFALS was detected in both patient groups and was prioritized for validation in the independent cohorts (Table S4). Notably, more unique protein hits were identified in the piME/CFS group (52) than in the pcME/CFS group (19), while a higher number of unique proteins were found in the pcHC group (41) than in the HC (31) group. As highlighted (red), only LYAM1/L-selectin was present in three out of nine patients in the pcME/CFS group (Figure 4A, green box). In contrast, several unique proteins were identified in at least three individuals of the piME/CFS group (Figure 4B, blue box), including AFAM/Afamin, APOH/ß2-glycoprotein-1, CO7/Complement factor 7, FA11/coagulation factor XI, KLKB1/plasma kallikrein, and a variable immunoglobulin chain (HV64D). Furthermore, only in the pcHC group, but not in the pre-pandemic HC group, were several proteins found in at least three participants, including CALL5/Calmodulin-like protein 5 and LYAM3/P-selectin, as well as several variable chains of immunoglobulins (HV309,348,373,439, KV439), as highlighted in Figure 4A (gray box).

**Figure 5:**
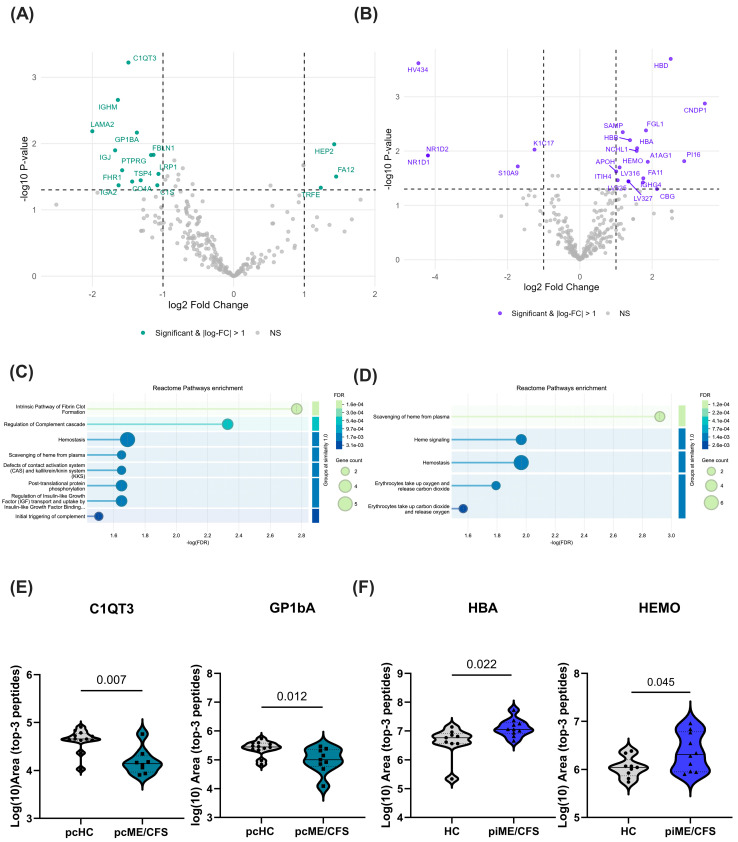
Differential EV protein signatures in ME/CFS cohorts. EV-associated proteins quantified by LC–MS from post-COVID-19 ME/CFS (pcME/CFS) and post-infectious ME/CFS (piME/CFS) patients and their respective healthy controls were analyzed using normalized label-free quantification intensities as described in the Methods. Volcano plots display log_2_ fold change versus −log_10_ (*p* value), with significance thresholds set at *p* < 0.05 and |log_2_ fold change| ≥ 1 (dashed lines), for (**A**) pcME/CFS versus pcHC and (**B**) piME/CFS versus pre-pandemic HC. Proteins meeting these criteria are highlighted and annotated with their UniProt identifiers. Reactome pathway enrichment based on significantly regulated proteins is shown for (**C**) pcME/CFS versus pcHC and (**D**) piME/CFS versus HC using STRING analysis, plotted as −log(FDR) at a similarity score of 1.0. (**E**) Semiquantitative analysis of normalized label-free quantification values (log_10_ area of the top three peptides) for C1QT3 and GP1bA in pcME/CFS versus pcHC, and (**F**) for HBA and HEMO in piME/CFS versus HC. Statistical significance was assessed using the Mann–Whitney U test (*n* = 9–12 per group).

Next, the complete set of EV proteins identified in both mass spectrometry analyses were examined after normalizing the datasets, as described in the Methods, resulting in 251 candidate proteins for the pcME/CFS vs. pcHC comparison and 295 candidates for the piME/CFS vs. pre-pandemic HC comparison. Limma analysis was applied to statistically determine the differentially regulated proteins between the patient groups and their respective HC groups. This comparative analysis of exploratory mass spectrometry data revealed 16 proteins that were significantly altered in the pcME/CFS group compared to pcHC (log_2_-fold change ≥ ±1, *p* < 0.05), as shown in the volcano plot (Figure 5A). Of these, three proteins were upregulated and thirteen were downregulated. In piME/CFS patients, 23 proteins were significantly different compared to the pre-pandemic HC group (Figure 5B), with 18 upregulated and 5 downregulated proteins. Potentially diagnostically relevant proteins—including Complement C1q tumor necrosis factor-related protein 3 (C1QT3), Glycoprotein Ib Platelet Subunit Alpha (GP1bA), Thrombospondin (TSP), Hemopexin (HEMO), and hemoglobin subunit alpha (HBA)—were selected for further validation by ELISA in an independent cohort and are listed with their names, logFC, and *p*-values in Supplementary Tables S5 and S6, respectively.

Analyzing the normalized data (top-3 peptide-area) from label-free quantification by mass spectrometry for all patients and HC samples, the differential abundance of the selected protein candidates was confirmed by visualizing the individual values in each study group (Figure 5E,F). As already shown in the volcano plots (Figure 5A), where the values for the proteins C1QT3 and GP1bA were significantly lower in the pcME/CFS study group compared to the pcHC group, the same significantly reduced levels were observed in the pcME/CFS group when examining the summarized top-3 peptide areas (Figure 5E). Similarly, proteins HBA and HEMO, which showed significant upregulation in the piME/CFS group compared with the corresponding HC group in the statistical analysis (Figure 5B), also exhibited significantly higher normalized LFQ values in the piME/CFS cohort when analyzing the top-3 peptide-area values (Figure 5F).

Analyzing the significantly regulated protein candidates from each of the two proteomic datasets in the STRING database for functional enrichment, we found that the majority of proteins belong to the following cellular compartments: blood microparticles, the extracellular space, the extracellular region, and extracellular exosomes (Supplementary Figure S2A,B). Interestingly, the most strongly enriched Reactome pathways differed between the pcME/CFS and piME/CFS proteomic studies (Figure 5C,D). In the pcME/CFS analysis, the main pathways were fibrin clot formation, regulation of the complement cascade, and hemostasias (Figure 5C), whereas in the piME/CFS setting, they included heme degradation, heme signaling, hemostasis, and oxygen uptake and carbon monoxide release by erythrocytes (Figure 5D).

### 2.4. A Selection of Identified Plasma EV Markers Shows Higher Plasma Levels in Validation Cohorts of Pime/CfS Patients Compared to HCS

Next, the expression of five selected protein candidates, namely HBA, HEMO, C1QT3, GP1bA, and TSP-1, as one member of the TSP family and a substitute for TSP-4, which were identified in the quantitative analysis (see Figure 5), and one protein, ALS/IGFALS, identified in the qualitative analysis (see Supplementary Table S4), was analyzed by ELISA in plasma samples to validate their potential as biomarkers.

**Figure 6:**
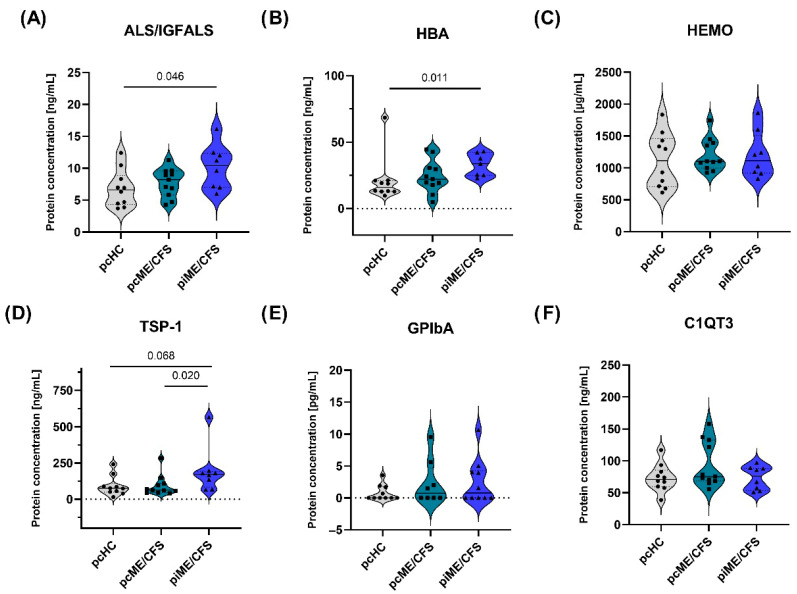
Plasma concentrations of selected protein candidates in ME/CFS groups. Plasma levels of candidate proteins were quantified by ELISA in post-COVID-19 ME/CFS (pcME/CFS), post-infectious ME/CFS (piME/CFS), and post-COVID healthy control (pcHC) cohorts. Violin plots show the median (solid line), interquartile range (dotted lines), and individual sample values for (**A**) ALS/IGFALS, (**B**) HBA, (**C**) HEMO, (**D**) TSP-1, (**E**) GP1bA, and (**F**) C1QT3. Protein concentrations are reported in µg/mL or ng/mL, depending on the analyte. Statistical significance was assessed using one-way ANOVA for panel (**A**), the Kruskal–Wallis test with Benjamini–Yekutieli–Krieger false discovery rate correction for panel (**B**), and the Mann–Whitney U test for panel (**D**). Sample sizes were pcME/CFS (*n* = 11), piME/CFS (*n* = 8), and pcHC (*n* = 10).

Plasma samples from the validation cohort (Table 1), consisting of pcME/CFS (*n* = 11), piME/CFS (*n* = 8), and pcHC (*n* = 10), were analyzed for the selected protein candidates (Figure 6). Significantly higher plasma concentrations of ALS/IGFALS (Figure 6A; *p* = 0.046) and HBA (Figure 6B; *p* = 0.011) were detected in the piME/CFS group compared to HC. TSP-1 concentrations tended (*p* = 0.068) to be higher in the piME/CFS group compared to the HCs and was significantly higher (*p* = 0.020) compared to pcME/CFS group (Figure 6D). Protein concentrations of the other candidates (HEMO, GP1bA, C1QT3) were similar between patients and HCs (Figure 6C–F). GP1bA, which was identified as a downregulated EV candidate in the proteomics analysis (Figure 5B), was detectable only in a subset of plasma samples across all study groups and did not show significant group differences (Figure 6E).

### 2.5. Identification of Significantly Regulated miRNAs in EV Cargo Associated with pcME/CFS and Correlation with Symptom Severity

**Figure 7:**
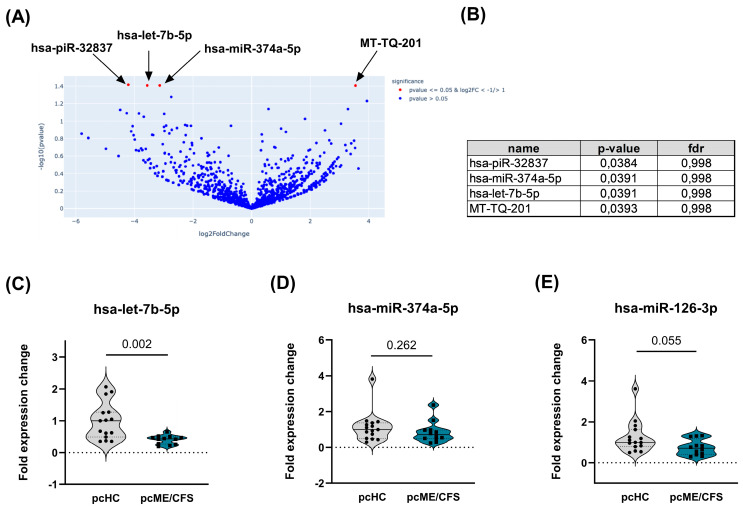
Identification and validation of differentially expressed EV-associated small RNAs in post-COVID-19 ME/CFS. (**A**) Volcano plot showing differential expression of small RNAs in EVs from post-COVID-19 ME/CFS (pcME/CFS) patients versus post-COVID healthy controls (pcHC) in the discovery cohort (*n* = 3 per group). The *x*-axis represents log_2_ fold change (pcME/CFS vs. pcHC) and the *y*-axis −log_10_(*p* value). Significantly differentially expressed RNAs are highlighted in red and annotated (hsa-piR-32837, hsa-let-7b-5p, hsa-miR-374a-5p, MT-TQ-201); non-significant RNAs are shown in blue. (**B**) Table of significantly differentially expressed small RNAs (*p* < 0.05), including *p*-values and false discovery rate (FDR).Validation by qPCR was performed as described in the Methods, and relative expression was calculated using the 2^−ΔΔCt^ method. Violin plots display the median (solid line), interquartile range (dotted lines), and individual values for (**C**) hsa-let-7b-5p, (**D**) hsa-miR-374a-5p, and (**E**) hsa-miR-126-3p. Statistical significance was assessed using the Mann–Whitney U test. Sample sizes were pcME/CFS (*n* = 12) and pcHC (*n* = 15).

In addition to the investigation of protein markers in the EV cargo, this study also aimed to characterize the miRNAome of isolated plasma EVs. Consequently, miRNA was isolated from EVs of pcHCs and pcME/CFS patients (*n* = 3/cohort), and RNA-seq was performed as described in the Methods section. On average, 3.3 × 10^6^ reads were sequenced in the pcHC samples and 2.8 × 10^6^ reads in the pcME/CFS samples. From these reads, an average of 117 miRNAs were mapped in the pcHC samples, while 91 miRNAs were successfully mapped in the pcME/CFS patients. In total, 55 miRNAs were identified across all samples and used for differential expression analysis between the two groups (Figure 7A). Four small RNAs were found to be significantly differentially expressed between the pcHC donors and the pcME/CFS patients (Figure 7B). MT-TQ-201 (log_2_ fold change = 3.5), a transfer RNA, was the only small RNA that was significantly upregulated in pcME/CFS patients (Figure 7A). In contrast, the two miRNAs hsa-miR-374a-5p (log_2_ fold change = −3.1) and has-let-7b-5p (log_2_ fold change = −3.6), as well as the Piwi-interacting RNA piR-32837 (log2 fold change = −4.2), were significantly downregulated in patients compared to the HC group (Figure 7A).

Subsequently, RNA was isolated from peak fractions of two SEC runs and cDNA was synthesized as described in the Methods section. Then, qPCRs were performed for the two significantly dysregulated miRNAs found in the RNA-seq experiment. In addition to hsa-miR-374a-5p and hsa-let-7b-5p, we further assessed the expression of hsa-miR-126-3p, previously reported to be altered in plasma from ME/CFS patients [45]. The exogenous spike-in ath-miR-159a was used to monitor technical quality; its stable expression across all samples indicated no extraction- or batch-related variation. Furthermore, hsa-miR-186-5p, used as the endogenous normalization control, displayed stable expression in all samples (). The qPCR results for the pcHC and pcME/CFS patient cohorts are shown as fold changes in expression for hsa-let-7b-5p (Figure 7C), hsa-miR-374a-5p (Figure 7D) and hsa-miR-126-3p (Figure 7E). Interestingly, a significantly lower expression of hsa-let-7b-5p was detected by qPCR in the pcME/CFS cohort compared to pcHCs (*p* = 0.002) (Figure 7C). Expression of hsa-miR-126-3p expression also tended to be lower (*p* = 0.055) in pcME/CFS patients compared to pcHCs (Figure 7E). This non-significant downward trend for hsa-miR-126-3p was also observed in the RNA-seq dataset (log_2_ fold change = –1.4; *p* = 0.297,). The hsa-miR-374a-5p expression level did not differ (*p* = 0.262) between pcME/CFS patients and pcHCs (Figure 7D).

**Figure 8:**
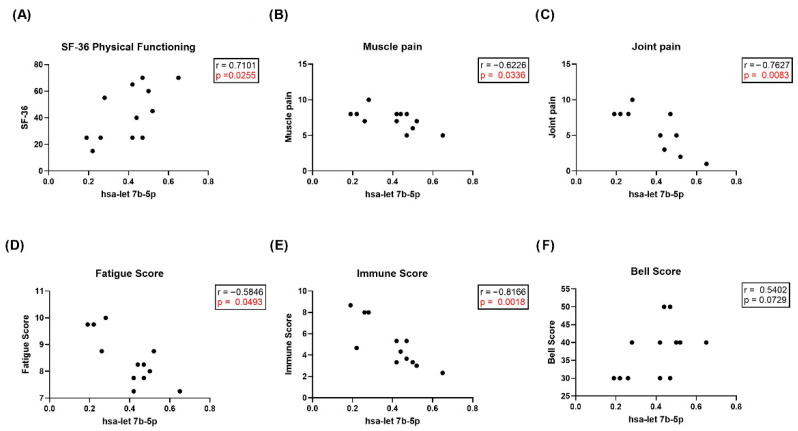
EV-associated hsa-let-7b-5p and clinical symptom severity in post-COVID-19 ME/CFS. Scatter plots show correlations between the relative expression of hsa-let-7b-5p and clinical scores in post-COVID-19 ME/CFS (pcME/CFS) patients for (**A**) SF-36 Physical Functioning, (**B**) muscle pain, (**C**) joint pain, (**D**) fatigue score, (**E**) immune score, and (**F**) Bell score. Each data point represents one patient (*n* = 10–12). Spearman’s rank correlation coefficients (ρ) and corresponding *p*-values are shown in the upper right corner of each plot, with statistically significant correlations highlighted in red.

In correlation analysis of qPCR results for all tested small RNAs with clinical symptom scores in the pcME/CFS validation cohort, we found a significant positive correlation (r = 0.7101; *p* = 0.0255) between the expression levels of hsa-let 7b-5p and physical functioning as assessed by the short form health survey 36 (SF-36) questionnaire (Figure 8A). In addition, significant negative correlations were observed between hsa-let-7b-5p expression and the severity of muscle pain, joint pain, fatigue, and immune symptoms (Figure 8B–E). Moreover, reduced hsa-let-7b-5p expression tended to correlate (r = 0.5402; *p* = 0.0729) with increased patient’s disability as assessed by the Bell score in the pcME/CFS validation cohort (Figure 8F). No correlations between miRNA expression and age or BMI were observed.

When analyzing the potential associations between the three miRNAs by correlation analysis (Figure S3), no significant correlations were detected between hsa-miR-374a-5p or hsa-miR-126-3p and hsa-let-7b-5p, nor between hsa-miR-126-3p and hsa-miR-374a-5p (Supplementary Figure S3A–C).

## 3. Discussion

In this study, we comparatively investigated EVs isolated from the plasma of post-infectious ME/CFS patients and HCs to characterize their protein and miRNA cargo profiles. In addition, identified EV protein and miRNA markers were correlated with clinical symptom severity. For the protein markers, ME/CFS patients were further categorized by the cause of their disease, differentiating between SARS-CoV-2 infection and other infectious triggers. Firstly, we isolated small EVs from the plasma of post-infectious patients using the highly reproducible SEC method and characterized them comprehensively. This allowed us to confirm that small EVs with typical size and morphology are released into the plasma by different types of cells. Moreover, the expression of canonical EV surface tetraspanins (CD9, CD63, and CD81) was confirmed via multiplex flow cytometry. This analysis revealed differences in the relative expression levels of the various tetraspanins. Among these, CD81 was consistently the most abundantly expressed, whereas CD9 and CD63 showed comparable but lower expression levels.

Moreover, our surface marker analysis similarly revealed only marginal differences between study groups. Interestingly, the only statistically significant differences involved CD9 and CD81 expression levels, which differed between EVs from HCs and piME/CFS patients, but not between pcME/CFS patients and pcHCs. The biological significance of increased CD81 on piME/CFS-derived EVs remains unclear. In the immune system, CD81 regulates the immunological synapse, the grouping of receptors in the cell membrane, and reduces the immune response [46]. CD9, which tended to be reduced on piME/CFS-derived EVs, is known as an anti-inflammatory marker of monocytes and IL-10 producing regulatory B cells [47]. Differences in tetraspanins levels have not been described in the context of ME/CFS to date, but may be worth monitoring in the future due to their known functional role in immune cells. Other EV markers originating from different cellular sources were found to differ in their abundance in post-infectious ME/CFS when EVs were analyzed directly in plasma via flow cytometry without prior isolation [48].

To date, no published study has used the MACSPlex Exosome Kit to profile the surface marker composition of plasma-derived extracellular vesicles in ME/CFS patients. Using this method, we could demonstrate the expression not only of tetraspanins, but also of markers originating from different cellular sources. Despite the comprehensive profiling, we observed only limited differences between patient and control EVs. A notable exception was integrin CD29, which showed significantly reduced expression on EVs from piME/CFS patients compared to HCs (Figure 3F). All other analyzed markers did not display any differential expression between the cohorts. In contrast, other studies have reported significant changes in EVs from patients with acute SARS-CoV-2 infection that could serve as indicators of infection severity [49]. Burrello et al. found high expression of CD4, CD45, and CD142 in deceased patients, with CD142 exhibiting the strongest correlation with disease prognosis. Another study found evidence that the sera of COVID-19 patients with mild infection contained higher levels of CD24-expressing EVs. Those EVs acted as indicators of mild infection, while severe COVID-19 patients showed higher levels of CD82-expressing EVs [50].

However, in our study of post-infectious ME/CFS patients, we could not identify a specific surface marker differentially expressed on plasma EVs. To obtain a thorough profile of protein alterations, we therefore investigated the EV proteome and applied two different independent mass spectrometry analyses for (1) pcME/CFS patients and (2) piME/CFS patients with their corresponding HCs. In each of our two exploratory EV proteome studies, we were able to detect a comparable number of candidate proteins. First, we performed a qualitative analysis using the STRING database, taking into account that not all proteins present in plasma EVs are abundant enough to be detected in the LC-MS analyses. Another argument in favor of an initial qualitative overview of the EV proteome is that we analyzed the proteome of an isolated small subpopulation of vesicles rather than the entire plasma. Therefore, we performed a stepwise selection based on existing knowledge of the EV source and the known pathomechanistic pathways/processes involved in ME/CFS, using an appropriate selection of STRING-based GO terms such as *cellular component*, *biological process*, and *subcellular localization*, as described in detail in the Methods section. This approach opened the possibility of identifying novel, interesting plasma EV-associated targets related to ME/CFS that may develop either after SARS-CoV-2 or after another type of infection.

Interestingly, ALS/IGFALS was consistently identified as a unique candidate in both piME/CFS and pcME/CFS patients compared to their corresponding HCs (Supplementary Table S4). This molecule has previously been described in the context of ME/CFS [35] in pre-pandemic EV and plasma samples from HCs and ME/CFS patients. Accordingly, we found significantly enhanced ALS/IGFALS levels in the piME/CFS cohort (Figure 6A), but not in the pcME/CFS cohort, resembling the findings of others [35]. This molecule, although largely under-recognized, warrants further investigation in the context of ME/CFS due to its involvement in immune processes, including antiviral signaling, and its suppression by interleukin-1 beta. It facilitates the formation of a complex between tumor necrosis factor, receptor-associated factor 6 and interleukin-1 receptor-associated kinase 1, thereby enabling subsequent activation of pathways for antiviral protein expression. The elevated ALS/IGFALS concentrations in plasma found in this study may reflect a consequence of the viral trigger for ME/CFS. Furthermore, its known role in the regulation of insulin-like growth factors 1 and 2, and consequently the regulation of metabolism, may be relevant in the context of ME/CFS. Other studies have shown that higher ALS/IGFALS levels correlate with higher immunoglobulin E levels in patients with childhood asthma, whereas reduced ALS/IGFALS levels were reported in autoimmune cirrhosis and acute COVID-19 patients.

By analyzing all proteins identified in the proteome analyses via FunRich, we found unique protein candidates for both the pcME/CFC vs. pcHC and piME/CFS vs. HC comparisons. However, only a few of these unique proteins were present in more than three samples within each group. L-selectin, which we identified in the pcME/CFS group, and P-selectin in the piME/CFS group are proteins previously discussed as serum markers in Long-COVID [51,52,53]. P-selectin, in particular, could contribute to endothelial dysregulation and thrombocyte activation in ME/CFS and Long-COVID. Interestingly, L-selectin may be involved in the solubility and activity of lipidated Wingless/Integrated signaling family members in body fluids [54]. Another candidate identified in the piME/CFS group is β2-glycoprotein-I, which binds negatively charged molecules, prevents activation of the intrinsic blood coagulation cascade, and is the primary antigen in the autoimmune disease antiphospholipid syndrome [55]. Future screening of patient sera could therefore include these proteins, specifically to test their association with post-infectious ME/CFS. In our exploratory quantitative EV proteome analysis comparing pcME/CFS with pcHCs, we identified 16 significantly up- or downregulated proteins (Figure 5A), while 23 proteins were found to be upregulated in the piME/CFS analysis compared to the corresponding HCs (Figure 5B). It is striking that both proteome analyses revealed distinct proteins associated with different Reactome pathways. These differences may be attributed to the considerably shorter disease duration in the pcME/CFS group (median 12 months; Supplementary Table S2) compared to the piME/CFS group (median 78 months; Supplementary Table S3). For example, proteins involved in complement activation and clot formation predominate in the pcME/CFS group, whereas proteins and pathways involved in heme binding and the maintenance of homeostasis and erythrocyte function dominated in the piME/CFS group. This divergence could be due to the fact that the pcME/CFS cohort was examined closer to the time of the initial trigger. Alternatively, distinct infectious triggers may cause altered protein expression and thus distinct Reactome profiles. This finding should be taken into account in future diagnostic analyses.

Interestingly, some of the identified proteins, such as HEMO and HBA, have also been reported in other studies [35,39,56,57]. While many studies stop at exploratory findings, we aimed to validate selected EV-associated candidate proteins in independent patient cohorts by ELISAs using plasma as an easily accessible source. By measuring HBA in plasma using an ELISA that detects both the unmodified α subunit and its glycated form, HbA1c, we found significantly elevated levels in piME/CFS patients compared to HCs (Figure 6B). Glycation of HBA occurs through non-enzymatic glucose modification and results in the formation of HbA1c—a standard clinical marker for long-term glucose homeostasis and diabetes management. Notably, elevated HbA1c has been associated with increased body pain and fatigue in type 1 diabetes patients [58,59]. Moreover, higher HbA1c plasma levels were recently documented in post-acute sequelae of COVID-19 (PACS) [60] and identified as a risk factor for developing Long-COVID in type 2 diabetic patients [61,62]. In this context, higher HbA1c levels were linked to a greater risk of respiratory symptoms and cognitive dysfunction, including brain fog [62]. However, in contrast to these findings, our study did not detect significantly enhanced HBA/HbA1c plasma level in the pcME/CFS cohort, suggesting that post-COVID and post-infectious ME/CFS may differ in their metabolic signatures or in the extent to which glucose dysregulation contributes to disease pathology. Although others have recently identified HEMO in the plasma proteome of post-COVID-19 patients as a promising biomarker within a set of four markers [63], showing nearly 10-fold higher expression in patients versus HCs, we could not validate a significant increase in HEMO levels by ELISA, despite the initial elevation observed in our EV proteome dataset for piME/CFS vs. HC (Figure 5B). Nevertheless, the occurrence of this marker in our proteome study identified it as a potentially relevant diagnostic candidate [39]. Giloteaux et al. [39] reported that the ratio of 24 h/15 min HEMO levels in EVs from ME/CFS patients after cardiopulmonary exercise testing was negatively correlated with SF-36 scores measuring health status. In another plasma proteome study by Cervia-Hasler et al. [57], lower HEMO level was identified in Long-COVID patients compared to HCs. Moreover, in this study, lower HEMO concentrations were associated with increased levels of circulating heme, which could promote oxidative damage [57]. Besides HEMO, haptoglobin, another heme-binding protein, has come into focus as a diagnostic marker for PEM in ME/CFS. Moezzi et al. observed that ME/CFS patients demonstrated a significant reduction in haptoglobin levels following post-exertional stress and that lower baseline concentrations were associated with impaired cognitive performance [64]. Moreover, another potential diagnostic marker found in other studies [57] is TSP-1, which showed increased plasma levels in patients. In our study, we observed a trend toward elevated plasma concentrations in the piME/CFS group compared to HCs (Figure 6D), and no elevation was detected in the pcME/CFS patient group. In a recent targeted plasma proteome study, Nunes et al. reported significantly elevated expression of TSP-1 in ME/CFS patients relative to HCs, with neither group having a history of SARS-CoV-2 infection [56]. Contractionary plasma proteome-based results have been recently described, with TSP-1 downregulated in Long-COVID patients versus HCs [65] or upregulated in acute COVID-19 and convalescent COVID-19 patients [66]. Thrombospondin-1, a multifunctional, matricellular, and highly glycosylated protein primarily released by platelets, can exert diverse biological effects through interactions with its receptors CD36 and CD47 [67]. Its anti-angiogenic activity may be relevant to post-infectious ME/CFS pathogenesis and potentially contribute to vascular dysfunction. In addition, its involvement in clot formation through incorporation into fibrin—recently described in Long-COVID—could promote thrombo-inflammatory alterations [68,69].

In summary, HBA was the only marker among the identified proteins in our quantitative analyses that could be validated by ELISA, exclusively in the piME/CFS cohort. Moreover, ALS/IGFALS, identified by our qualitative approach, also displayed significantly enhanced levels only in the piME/CFS cohort. However, to reliably assess the clinical significance of these findings, validation in larger and well- characterized patient cohorts will be essential.

To explore further potential diagnostic biomarkers for ME/CFS, we investigated the miRNA cargo of plasma EVs. First, we conducted an RNA sequencing analysis focused exclusively on the pcME/CFS cohort and the corresponding pcHC group. Among the differentially expressed candidates, the two miRNAs that were significantly downregulated in this sequencing approach, namely hsa-miR-374a-5p and hsa-let-7b-5p (Figure 7), were tested in a larger validation cohort. While we observed no significant differences between pcME/CFS and pcHC groups for hsa-miR374a-5p, a significantly lower level was found for hsa-let-7b-5p in the patient group. Hsa-let-7b-5p emerges as a novel miRNA of interest, as it has not previously been reported in ME/CFS or Long-COVID. Within the let-7 family, only hsa-let-7d-5p has been described in EVs from ME/CFS patients, where it was upregulated compared to HCs [41]. In contrast, hsa-let-7b-5p appears biologically more complex. Evidence from autoimmune disease suggests an anti-inflammatory function. For instance, reduced expression in the cerebrospinal fluid correlates with increased inflammation and clinical severity in multiple sclerosis, supporting its putative role as a suppressor of inflammatory pathways [70]. However, in rheumatoid arthritis, hsa-let-7b-5p has been proposed to act pro-inflammatory within synovial EVs, with transforming growth factor beta receptor 1 and insulin-like growth factor 1 receptor identified as candidate downstream mediators [71]. Moreover, reduced EV-associated hsa-let-7b-5p expression has been documented in psoriatic arthritis, where lower levels correlate with more severe clinical presentation [72]. The function of this miRNA appears to be dependent on the specific target molecule, as a pro-inflammatory role has been reported through Toll-like receptor 7 activation in neurodegeneration [73] and in rheumatoid arthritis [74].

We hypothesize that in our pcME/CFS patients, the reduced expression of hsa-let-7b-5p may contribute to an insufficiently regulated immune response and persistent low-grade inflammation. This assumption is supported by the strong and statistically significant correlations found with clinical symptom scores (Figure 8), most prominently its negative association with the severity of immune symptoms (Figure 8A). The negative correlations with the severity of muscle and joint pain (Figure 8B,C) are consistent with previous suggestions of tissue-specific miRNA release into plasma EVs [41,75]. The markedly lower expression of hsa-let-7b-5p in human plasma EVs could serve as a valuable and promising biomarker candidate for piME/CFS, at least for the subgroup of ME/CFS patients triggered by an SARS-CoV-2 infection. Interestingly, hsa-let-7b-5p has also been found to be significantly reduced in the nasopharyngeal swabs of acute COVID-19 patients [76]. In line with this, it has been demonstrated that hsa-let-7b-5p regulates genes such as angiotensin-converting enzyme 2 and dipeptidyl peptidase-4, both required for SARS-CoV-2 entry. Whether hsa-let-7b-5p downregulation also occurs in ME/CFS patients with other infectious trigger remains unknown and should be investigated in future studies. Several other differentially expressed miRNAs have been described in ME/CFS patients—for instance hsa-miR-127-3p, hsa-miR-140-5p, and hsa-miR-374b-5p—which were found to be upregulated in plasma samples of patients compared to HCs [41,42,77,78]. In contrast, hsa-miR374b-5p levels were significantly downregulated as measured by RNA sequencing and showed a downward trend when measured by qPCR in independent pcME/CFS cohorts compared to pcHCs (Figure 7D). An involvement of this miRNA is supported by its established multifaceted immunological functions, including its capacity to inhibit neuroinflammation via NLR family pyrin domain containing 3 inflammasome regulation [79], its role as a modulator of inflammation in obesity [80], and its ability to regulate inflammatory gene expression and monocyte activity in inflammatory bowel disease [81]. Thus, the involvement of hsa-miR-374b-5p in the pathogenesis of post-infectious ME/CFS remains a biologically plausible hypothesis and warrants deeper investigation.

Another miRNA candidate, hsa-miR-126-3p, has previously been reported as differentially expressed in the plasma of ME/CFS [15,45,82,83] and Long-COVID [84] patients. Hsa-miR-126-3p is a key regulator of endothelial biology, capable of enhancing VEGF signaling via suppression of two negative pathway regulators [85]. In our present study, we observed reduced hsa-miR126-3p expression in pcME/CFS patients compared to HCs, although this was also only observed as a trend (Figure 7E). This aligns with findings by Cheema et al. [84]. However, other studies reported a significant upregulation of this miRNA in mild or severely affected ME/CFS cohorts [41,45,82]. Differences in patient characteristics, disease phase, or infectious triggers may underlie these divergent observations. In our experiment, we focused exclusively on female patients whose ME/CFS was triggered by COVID-19, factors that may contribute to the observed response pattern.

Despite the identification of several promising markers for post-infectious ME/CFS in this study, certain limitations must be acknowledged, particularly the relatively small sample sizes in both the exploratory groups and validation cohorts. This limited size may affect the detection of protein markers in plasma as well as the assessment of miRNA expression in isolated EVs. In addition, the time passed since the initial SARS-CoV-2 infection differed between pcME/CFS and pcHC individuals in the proteome exploration cohort. Nevertheless, despite the limited number of patients included in the miRNA analyses, we demonstrated robust and statistically significant correlations between hsa-let-7b-5p expression levels and multiple clinical symptom scores within the pcME/CFS cohort. These findings support the role of hsa-let-7b-5p as a strong molecular candidate associated with clinical disease severity in this patient population. Moreover, given the relatively small sample size of the validation cohorts, only the miRNA candidates could be correlated with clinical scores and symptom parameters. For the identified protein markers, correlation analyses in substantially larger patient cohorts will be required, along with a systematic evaluation of diagnostic specificity and sensitivity, either for individual proteins or marker combinations. Furthermore, miRNA detection by qPCR was performed using a limited subset of miRNAs identified in our initial RNA sequencing screen. However, the initial correlation analysis with clinical symptom scores was successful and provides indicative evidence for the potential pathomechanistic involvement of these candidates in ME/CFS.

## 4. Material and Methods

### 4.1. Study Cohorts/Patients

All patients were diagnosed with ME/CFS based on the Canadian Consensus Criteria at the Charité Fatigue Centre [86]. Patients included in this study developed ME/CFS either following SARS-CoV-2 infection (pcME/CFS) or after another non-SARS-CoV-2 infection (piME/CFS). All participants signed an informed consent form prior to study enrolment. All studies in this patient cohort were approved by the ethics committee of Charité Universitätsmedizin Berlin, Germany (EA2/067/20, EA2/066/20, EA2/070/20), following the 1964 Declaration of Helsinki and its subsequent amendments.

Plasma from piME/CFS and HCs (without self-reported SARS-CoV-2 infection) were collected between 2020 and 2021. Plasma samples from HC with previous self-reported SARS-CoV-2 infection (referred to as pcHC), as well as from pcME/CFS patients, were collected between 2022 and 2023.

Detailed cohort information is displayed in Table 1, Table 2 and Tables S1–S3. To reduce biological heterogeneity in this exploratory study, only female participants were included, reflecting the strong female predominance of ME/CFS. Severity of disease and symptoms were evaluated in patients with appropriate questionnaires. The functional disability was assessed using the Bell score, ranging from 0 to 100 (100 indicating no restrictions) [87], and physical function was evaluated using the SF-36, ranging from 0 to 100 (higher scores indicating better function) [88]. The severity of the key symptoms, fatigue, pain, cognitive impairment and immune symptoms, was quantified using a Likert scale (1 = no symptoms to 10 = severe symptoms). PEM severity was assessed according to the brief DePaul Symptom Questionnaire—post-exertional malaise, with scores ranging from 0 to 46 (higher scores indicating more frequent/severe PEM) [89]. Autonomic dysfunction was assessed using the Composite Autonomic Symptom Score 31, ranging from 0 to 100 (higher scores indicating stronger impairment) [90]. Study data, including clinical parameters, were collected and managed using REDCap electronic data capture tools (version 15.5.35, Vanderbilt University, Nashville, TN, USA) hosted at Charité Universitätsmedizin Berlin [91,92].

### 4.2. Sample Collection and Preparation of Platelet-Free Plasma

Patient and HC blood samples were collected in ACD tubes (Greiner Bio-One GmbH, Kremsmünster, Germany). Within one hour of collection, the blood samples were centrifuged for 15 min at 2500× *g*. The plasma was then transferred into a new tube and centrifuged again for 15 min at 2500× *g*. Subsequently, the plasma was collected and stored at −80 °C until further use.

### 4.3. Size-Exclusion Chromatography (SEC) for Isolation of EVs

SEC was performed to isolate plasma EVs using the IZON automatic fraction collector and compatible qEVoriginal/35 nm columns (Izon Science Ltd., Christchurch, New Zealand). Each column was used for a maximum of ten EV collections, and different columns were used for each individual study cohort. The IZON automatic fraction collector was prepared for collection by washing the qEVoriginal/35 nm column with 10 mL 0.5 M NaOH (Merck, Darmstadt, Germany) and subsequently equilibrating the column with 20 mL of 1× Dulbecco’s phosphate-buffered saline (DPBS) (Thermo Fisher Scientific, Waltham, MA, USA). Washing and equilibration were performed similarly after each sample isolation. Then, 1 mL plasma sample was thawed and centrifuged at room temperature at 2500× *g* for 10 min. After transferring the supernatant to a new tube, it was centrifuged again at 4 °C at 4500× *g* for 30 min. The supernatant was filtered through a 0.22 µm syringe filter (Sartorius, Göttingen, Germany) and then transferred into a low-binding tube (Sarstedt, Nümbrecht, Germany). The column was then loaded with 500 µL of filtered plasma, and the isolation process was started. During collection, 6 mL of 1× DPBS was added as running buffer for the isolation process. Six fractions with a volume of 500 µL each were collected into low-binding tubes and stored at −80 °C for further use.

### 4.4. EV Quantification by Nanoparticle Tracking Analysis (NTA)

The ZetaView Particle Tracking Analyzer equipped with ZetaView software (version 8.05.16 SP3, Particle Metrix, Inning, Germany) was used to determine the size and concentration of EV fractions after initial calibration (100 nm solution). The EV fractions were diluted with 1× DPBS to dilutions ranging from 1:500 to 1:4000. The dilutions were dependent on the optimal particle range of the instrument (10–1000 nm). Nine distinct camera positions were used to optically track and count the particles. The mean particle concentration (p/cm^3^) was automatically calculated and multiplied by the corresponding dilution factor. Moreover, the size of the particles was determined using calibration beads as reference.

### 4.5. Transmission Electron Microscopy (TEM)

EVs from the SEC peak fraction were positive-negatively stained as already described [93] and evaluated for morphology and diameter by TEM at the electron microscopy facility of Charité-Universitätsmedizin Berlin. Briefly, 15 μL of the EV sample was placed for 20 min on formvar-carbon-coated copper EM grids (Electron Microscopy Sciences, Hatfield, PA, USA). For fixation, the grids were incubated with 2% paraformaldehyde (Carl Roth, Karlsruhe, Germany) in 0.2 M PBS for 20 min, followed by incubation with 1% glutaraldehyde (Sigma-Aldrich, St. Louis, MO, USA) in 0.2 M PBS for 5 min. Hereafter, the grids were washed six times with distilled water. After the last washing step, the grids were incubated with 30 μL of 4% uranyl acetate and 2% methylcellulose (both Sigma-Aldrich, St. Louis, MO, USA) for 10 min at 4 °C. The grids were picked up with an eyelet, excess liquid was sucked off using filter paper, and the grids were allowed to dry overnight in the dark. After complete drying, the grids were carefully removed from the eyelet and stored in a grid box. Samples were examined by a Zeiss Leo 906 transmission electron microscope (Carl Zeiss Microscopy GmbH, Jena, Germany).

### 4.6. Bicinchoninic Acid (BCA) Assay for Measurement of Protein Concentration

All collected EV-containing pooled SEC fractions (F2–F4) were analyzed for protein concentration using the Pierce BCA Protein Assay Kit (Thermo Fisher Scientific, Waltham, MA, USA) according to the manufacturer’s instructions for the detection range 25–2000 µg/mL. All samples were tested in duplicate in a 96-well plate (Thermo Fisher Scientific, Waltham, MA, USA). Absorbance was measured using a Tecan Spark plate reader (Tecan Group Ltd., Männedorf, Switzerland) at 562 nm.

### 4.7. Characterization of EV Surface Markers by MACSPlex

In order to characterize the surface epitopes of isolated EVs, the *MACSPlex EV kit for immune-oncology, human* from Miltenyi (Miltenyi Biotec, Bergisch Gladbach, Germany) was used according to the manufacturer’s instructions. This kit is recommended under the MISEV guidelines [94]. Briefly, 30–45 μL, corresponding to 5–10 μg protein of pooled EVs, were diluted to 120 μL using MACSPlex buffer in low-binding tubes (Sarstedt, Nümbrecht, Germany). As a negative control, 120 μL of the buffer was used. Then, 15 μL of Exosome Capture beads was added to each tube and incubated overnight on a tube rotator under continuous motion. All samples were measured using a CytoFlex LX flow cytometer (Beckman Coulter Life Science, Brea, CA, USA) within one hour and analyzed using FlowJo (version 10, BD Life Sciences, Ashland, OR, USA).

The relative abundance of each surface marker was calculated as follows: First, the median fluorescence signal intensity (MFI) of each bead population from the blank control was subtracted from the corresponding MFI in each sample. Then, a normalization factor was determined by calculating the median intensity of the three tetraspanins CD9, CD63, and CD81 for each sample. Finally, the signal intensity of each bead population was divided by the sample-specific normalization factor to determine the relative expression level of each marker.

### 4.8. Proteomic Characterization of EV

Differentially expressed proteins in EVs from patients and HCs were detected using liquid chromatography–mass spectrometry (LC-MS). This was performed in two independent proteome analyses: one comparing pcME/CFS patients to their matched pcHCs, and another comparing the piME/CFS cohort with their corresponding controls, according to the procedure previously described in [93]. Cohort characteristics are displayed in Supplementary Tables S1 and S2.

#### 4.8.1. Sample Preparation

EV samples were prepared for proteomics analyses using the filter-aided sample preparation method [95]. Briefly, samples were mixed with 200 µL of 8 M urea in 0.1 mM Tris-HCl (pH 8.5; Sigma-Aldrich) and incubated for 10 min at room temperature. On-filter digestion was carried out using sequencing-grade modified porcine trypsin (20 µg; Promega Corporation, Madison, WI, USA) in 800 µL of 50 mM ammonium bicarbonate (Sigma-Aldrich). Digestion was performed in Amicon Ultra centrifugal filter units with a 10 kDa molecular weight cutoff (Merck Chemicals GmbH, Darmstadt, Germany) and incubated overnight at 37 °C. The resulting peptide mixtures were desalted using ZipTip C18 pipette tips (Merck Chemicals GmbH) according to the manufacturer’s instructions.

#### 4.8.2. Mass Spectrometry Analyses

For mass spectrometric analysis, 2 µL of each peptide extract eluate was injected into a high-performance liquid chromatography (nano-HPLC) system (Dionex Ultimate 3000, Thermo Fisher Scientific, Waltham, MA, USA). Peptide separation was performed using a 60 min gradient of 2–44% acetonitrile in 0.1% formic acid at a flow rate of 300 nL/min, employing a pre-column (trap column) and an analytical C18 column. The HPLC system was coupled to an ESI-QTOF mass spectrometer Impact II (Bruker Daltonik, Bremen, Germany) via a CaptiveSpray ion source. The ionization voltage was set to 1600 V, and the source temperature was maintained at 150 °C. Data acquisition was carried out in data-dependent acquisition (DDA) mode using the Instant Expertise method. The scan range was 150–2200 Da. Spectra were acquired at a rate of 2 Hz in MS mode and at 4–16 kHz in MS/MS mode, with a cycle time of 3 s in the QTOF reflector analyzer.

#### 4.8.3. Protein Identification

Protein identification was performed using PEAKS Studio software (version 10.6, Bioinformatics Solutions, Waterloo, ON, Canada). The generated peptide lists were searched against human entries in the UniProt database. Additionally, PEAKS de novo sequencing and the post-translational modification search tool were used to detect peptides not explicitly listed in the database but homologous to known sequences. A maximum of three variable post-translational modifications per peptide was allowed. Retention time shift tolerance was set to one minute. All search tools were integrated within the PEAKS X Pro platform. The false discovery rate was estimated using the target–decoy fusion method and set to 1%. Analysis parameters were set as follows: taxonomy restricted to Homo sapiens (20,175 sequences), trypsin as the proteolytic enzyme with a maximum of one missed cleavage, monoisotopic mass values, a peptide mass tolerance of 10 ppm, a fragment mass tolerance of 0.05 Da, and oxidation set as a variable modification. Significance filters were applied at −lgP > 20 for peptides and at −lgP > 15 for proteins, with the unique peptide filter set to 1.

### 4.9. Proteomic Data Processing

Proteins selected for downstream analysis were first identified using a qualitative approach. All proteins significantly detected (−lgP > 20 and a protein significance filter of −lgP > 15) in at least one sample from the patient groups (piMECFS and pcME/CFS) and their corresponding healthy donors were subjected to functional annotation using the STRING database (European Molecular Biology Laboratory & University of Zurich, Zurich, Switzerland). To systematically categorize these proteins, STRING enrichment analysis was performed across “GO” terms for “Cellular Component”, “Biological Process”, and “Subcellular Localization”. For each category, the enriched GO terms and their associated proteins were exported to Microsoft Excel (version 365, Microsoft Corporation, Redmond, WA, USA) and manually screened. Inclusion criteria focused on biological relevance to extracellular vesicles, endothelial or muscle biology, immune regulation, coagulation, angiogenesis, inflammation, and responses to oxidative stress. Within the “Subcellular Localization” category, proteins were prioritized based on GO terms associated with vesicles, exosomes, the cell surface, the complement system, actin-related structures, and platelets. Proteins not assigned to relevant GO terms in earlier rounds were carried forward and re-evaluated at each subsequent step. After these three enrichment rounds, a refined list of proteins was generated for each ME/CFS group (pcME/CFS and piME/CFS) and their matched healthy donors, identifying unique protein hits after the removal of overlapping entries. In parallel, VENN-analysis using FunRich software (version 3.1.3, La Trobe University, Melbourne, VIC, Australia) [96,97] was performed on all EV proteins initially found by LC-MS analysis to identify proteins present exclusively in one study group or shared between both groups within each proteomic dataset.

Label-free quantification was performed using PEAKS Studio software (version 10.6, Bioinformatics Solutions, Waterloo, ON, Canada), with automatic detection of the reference-sample and alignment of sample runs. The protein significance filter was adjusted to 1, the unique peptide filter was maintained at 1, and the protein fold change filter was set to 1. Finally, PEAKS analysis was conducted to identify differentially abundant proteins (significance mode PEAKSQ) using total ion count normalization.

To identify differentially expressed proteins between piME/CFS and HC cohorts, the normalized proteomic data were log_2_-transformed to approximate normality and reduce heteroscedasticity. A linear model was fitted to the transformed expression data, and empirical Bayes moderation was applied to obtain moderated t-statistics and adjusted *p*-values. Differentially expressed proteins were ranked accordingly. Volcano plots were generated to visualize log_2_ fold changes versus −log_10_(*p*) values, with statistical significance thresholds set at *p* < 0.05 and |log_2_ fold change| ≥ 1. Proteins exceeding these thresholds were highlighted in color.

### 4.10. Validation of Candidate Proteins with ELISA Systems

Plasma or EVs from patient cohorts and HCs were analyzed using commercial ELISA kits according to the manufacturer’s instructions for the following analytes: thrombospondin-1 (R&D Systems, Minneapolis, MN, USA), HEMO (Thermo Fisher Scientific, Waltham, MA, USA), GP1bA (MyBioSource, San Diego, CA, USA), ALS/IGFALS (BioLegend, San Diego, CA, USA), C1QT3 (Biolegend, San Diego, CA, USA) and HBA (Abcam, Cambridge, UK). However, due to insufficient assay sensitivity for detecting these proteins in isolated EV fractions, all measurements were ultimately performed using plasma samples. Samples and standard curves were run in duplicate and diluted as instructed by the manuals. Absorbance at 450 nm was recorded using a Tecan SPARK plate reader (Tecan Group Ltd., Männedorf, Switzerland). Protein concentrations were calculated from the respective standard curves and adjusted by appropriate dilution factors.

### 4.11. miRNA Isolation and Sequencing

For miRNA isolation, EV-containing fractions (F2–F4) from two independent SEC runs per donor were pooled and transferred into ultracentrifugation tubes. After adding 33 mL of DPBS, samples were centrifuged at 29,000 rpm for 2:45 h at 4 °C using an Optima L-80XP Ultracentrifuge (Beckman Coulter, Brea, CA, USA). The resulting EV pellet was resuspended in 700 μL QIAzol Lysis Reagent (Qiagen, Hilden, Germany) together with 4 μL of spike-in control (ath-miR-159a, 1 fmol/μL). Pellets were homogenized for 30 s using a T25 digital ULTRA-TURRAX homogenizer (IKA-Werke GmbH & Co. KG, Staufen, Germany). Total RNA was extracted using two phenol–chloroform extractions, after which the aqueous phase was mixed with 1.5 volumes of 100% ethanol (Serva Electrophoresis GmbH, Heidelberg, Germany) and loaded onto spin columns. miRNAs were isolated via the RNA Clean & Concentrator-5′ Kit (Zymo Research, Irvine, CA, USA). Following RNA isolation, samples underwent a final cleanup according to the manufacturer’s protocol optimized for retention of small RNAs (>17 nt), ensuring recovery of miRNAs within the expected size range (~20–24 nt) and suitability for downstream small RNA sequencing. Finally, RNA concentration and purity were assessed with a Nanodrop ND-1000 Spectrophotometer (Thermo Fisher Scientific, Waltham, MA, USA) at 230, 260 and 280 nm. Library preparation, sequencing and downstream bioinformatics analysis were performed for three RNA samples per group by the company GenXPro (Frankfurt am Main, Germany) using the ultra-sensitive TrueQuant small RNA-Seq kit. Sequences were produced using an NextSeq 500 system (Illumina, SanDiego, CA, USA) with 1 × 75 base pairs. Deduplication based on unique molecular identifiers was carried out using in-house scripts. Unprocessed sequencing reads underwent adapter and quality trimming using Cutadapt (version 4.3, Max Planck Institute for Developmental Biology, Tübingen, Germany). Quality assessment of the sequenced reads was performed with FastQC (Version 0.11.9, Babraham Bioinformatics, Cambridge, UK). Processed sequencing reads were aligned to the human reference genome using Bowtie2 (version 2.4.4, Johns Hopkins University, Baltimore, MD, USA) [98] and alignments were analyzed by Samtools (version 1.19/1.14, Wellcome Sanger Institute, Hinxton, UK). Aggregated sequencing QC software (MultiQC-version 1.16, SciLifeLab, Stockholm, Sweden) was used for aggregated quality assessment and differential expression analysis was performed using DE-Seq2. Targets were identified as differentially expressed upon a log2 fold change of −1< or >1 and a *p* value < 0.05. Results were compiled and visualized by GenXPro in a summary report.

### 4.12. cDNA Synthesis and Real-Time Quantitative PCR for Selected miRNAs

For cDNA synthesis, isolated miRNAs were reverse-transcribed using the TaqMan Advanced miRNA cDNA Synthesis-Kit (Thermo Fisher Scientific, Waltham, MA, USA) according to the manufacturer’s instructions. The resulting cDNA templates were analyzed by qPCR using TaqMan Advanced miRNA Assays (Thermo Fisher Scientific, Waltham, MA, USA). The following miRNA targets were selected for analysis: hsa-miR-126-3p, hsa-miR-374a-5p, and hsa-let-7b-5p, with hsa-miR-186-5p serving as the endogenous control and ath-miR-159a as the exogenous spike-in control. All qPCR samples were run in technical triplicates on a CFX Duet Real-Time PCR System (Bio-Rad Laboratories, Inc. Hercules, CA, USA). The 2^−ΔΔCt^ method was used to determine fold expression changes. Briefly, individual Ct values for each miRNA from every sample were normalized to the endogenous control: ΔCt = CtmiR–Cthsa-miR-186-5p. Then, ΔΔCt was calculated by normalizing to the median of HC samples: ΔΔCt = ΔCt–median ΔCtHC. Fold expression changes were calculated as 2^−ΔCt^.

### 4.13. Statistical Analysis

Statistical analyses were performed using GraphPad Prism Version 9.1.2 (GraphPad Software, San Diego, CA, USA) and R package (version 4.5.1, R Foundation for Statistical Computing, Vienna, Austria).

Patient characteristics are reported as median and range for continuous variables stratified by study group. Univariate comparisons of independent groups were performed using the Kruskal–Wallis test with a two-tailed *p* value ≤ 0.05. For comparisons between two independent groups, either an unpaired *t*-test (normally distributed data) or a Mann–Whitney U test (non-normally distributed data) was applied. All datasets were first evaluated descriptively, and mean values with interquartile ranges (IQRs) were calculated from untransformed and background-corrected data (protein ELISA) or normalized values (MACSplex, qPCR values for miRNA). For the LC-MS dataset, individual sample values were visualized as log_10_ transformed peptide areas (top-3-peptides) to improve scaling. Normality of MACSplex, ELISA and qPCR data was assessed using the Shapiro–Wilk and D’Agostino and Pearson tests. Comparative analyses of quantitative variables were performed using one-way ANOVA test without pairing for normally distributed data or a Kruskal–Wallis test with Benjamini, Krieger, and Yekutieli FDR correction for non-parametric data and multiple pairwise comparisons. A q-value ≤ 0.05 was considered as statistically significant. For two group comparisons, the Mann–Whitney U test was applied. As the majority of variables did not follow a normal distribution, Spearman correlation analysis was performed. Unless otherwise specified, two-tailed *p*-values ≤ 0.05 were regarded as statistically significant.

## 5. Conclusions

In this study, we identified potential diagnostic markers in the proteome and miRNA cargo of plasma-derived extracellular vesicles from two post-infectious ME/CFS cohorts: those triggered by SARS-CoV-2 (pcME/CFS) and those triggered by other infections (piME/CFS). The proteomic markers ALS/IGFALS and HBA were significantly elevated exclusively in the piME/CFS group, indicating that marker profiles differ depending on the initial disease trigger. This suggests that multi-analyte panels, rather than a single universal marker, may be required. In contrast, EV-associated miRNAs provided more robust and clinically meaningful results in pcME/CFS, with hsa-let-7b-5p emerging as a particularly strong candidate. It was significantly downregulated compared to HCs and correlated with multiple clinical severity parameters. This positions hsa-let-7b-5p as a promising diagnostic and pathophysiological marker specifically for COVID-19-associated ME/CFS. Testing this miRNA in additional piME/CFS cohorts will now be essential to determine whether its biomarker potential extends across the disease regardless of the infectious trigger. Overall, the integration of proteomic and miRNA profiles from EVs represents a compelling strategy to enhance diagnostic sensitivity, stratify patient subgroups, and advance the mechanistic understanding of piME/CFS. With the expansion of these findings to larger, well-characterized cohorts, these approaches could lay the groundwork for improved diagnostics and the development of targeted therapeutics.

## Figures and Tables

**Table 1:** Characteristics of the study groups (pcME/CFS, piME/CFS, pcHC) included in the protein marker validation. The median (interquartile range) of various parameters within the respective study group were presented. Statistical analysis was performed by Mann–Whitney U test (*p*-values) for the comparison of two groups and the Kruskal–Wallis test (q-values) for comparisons between all three groups. NA = not applicable; NI = no information.

	pcME/CFS (*n* = 11) Median with IQR	piME/CFS (*n* = 8) Median with IQR	pcHC (*n* = 10) Median with IQR	*p*-Value/ *q*-Value
Age [years]	36 (29–42)	25 (21–35)	33.5 (32–37)	0.06 ^a^ 0.63 ^b^ 0.11^c^
Female sex [%]	100	100	100	NA
BMI	23.1 (22.3–24.3)	21 (19.1–28.3)	NI	0.36
disease duration/ time since last SARS-CoV-2 infection [months]	12 (9.5–22.5)	24.5 (12–54)	10 * (5–14) ^§^	0.24 ^a^ 0.37 ^b^ 0.09 ^c^
Bell Score	40 (30–40)	40 (30–40)	NA	>0.99
SF-36 physical functioning	35 (30–60)	47.5 (41.3–57.5)	NA	0.45
Symptom severity scores:				
Fatigue Score	8 (6–8)	8.3 (6.4–8.8)	NA	0.50
Immune Score	3.3 (2.3–4.8)	4.7 (2.8–7.6)	NA	0.28
Cognitive Score	6.2 (4.2–7.7)	6.7 (4.5–8)	NA	0.68
Muscle Pain	6 (5–8)	7.5 (4.8–8)	NA	0.54
Joint Pain	5 (1–7)	6 (3.3–6.8)	NA	0.70
PEM score	34 (26–38)	35.5 (24.5–36.8)	NA	0.89

^§^ Information on self-reported SARS-CoV-2 infection was available from 7/10 of the HCs; * Time since last SARS-CoV2 infection; a = pcMECFS vs. piME/CFS; b = pc/MECFS vs. pcHC; c = piME/CFS vs. pcHC.

**Table 2:** Characteristics of the study groups included in the miRNA marker validation (pcME/CFS, pcHC). The median (interquartile range) of various parameters within the respective study group is presented. Statistical analysis was performed by Mann–Whitney test. NA = not applicable.

	pcME/CFS (*n* = 12) Median with IQR	pcHC (*n* = 15) Median with IQR	*p*-Value
Age [years]	39.5 (34–49)	37 (33–45)	0.28
Female sex [%]	100	100	NA
Disease duration/ Time since last SARS-CoV-2 infection [months]	14 (10–21)	11 * (8–13.3) ^§^	0.10
Bell Score	40 (30–40)	NA	NA
SF36 physical functioning	42.5 (25–63.8)	NA	NA
Symptom severity scores:			
Fatigue score	8 (7.8–9.5)	NA	NA
Immune score	4.5 (3.3–7.3)	NA	NA
Cognitive score	7.5 (6.1–8)	NA	NA
Muscle Pain	7.5 (6.3–8)	NA	NA
Joint Pain	5 (3–8)	NA	NA
PEM score	34 (30–41)	NA	NA

^§^ Information on self-reported SARS-CoV-2 infection was available from 12/15 of the pcHCs; * Time since last SARS-CoV-2 infection.

## Data Availability

The raw and processed miRNA expression data generated during this study have been deposited in the NCBI Gene Expression Omnibus (GEO) under accession number GSE317067. The mass spectrometry proteomics data have been deposited to the ProteomeXchange Consortium via the PRIDE [99] partner repository with the dataset identifier PXD073644.

## Supplementary Materials

The following supporting information can be downloaded at: https://www.mdpi.com/article/10.3390/ijms27052314/s1.

## Abbreviations

The following abbreviations are used in this manuscript:

ALS/IGFALS	Insulin-like growth factor acid labile subunit
C1QT3	Complement C1q tumor necrosis factor-related protein 3
DPBS	Dulbecco’s phosphate-buffered saline
ELISA	Enzyme-linked immunosorbent assay
EVs	Extracellular vescicles
FDR	False discovery rate
GP1bA	Glycoprotein Ib platelet subunit alpha
HbA1c	Glycated hemoglobin A1c
HBA	Hemoglobin subunit alpha
HC	Healthy control
HEMO	Hemopexin
IQR	Interquartile range
ME/CFS	Myalgic Encephalomyelitis/Chronic Fatigue Syndrome
MFI	Median fluorescence intensity
NTA	Nanoparticle tracking analysis
PACS	Post-acute sequelae of COVID-19
PCS	Post-COVID syndrome
PEM	Post-exertional malaise
SARS-CoV-2	Severe acute respiratory syndrome coronavirus 2
SEC	Size-exclusion chromatography
TEM	Transmission electron microscopy
