# Supplementary figures and images for: Extracellular Vesicle Protein and MiRNA Signatures as Biomarkers for Post-Infectious ME/CFS Patients
Source: Int J Mol Sci. 2026 Feb 28;27(5):2314. doi: 10.3390/ijms27052314 (PMC12984851; doi:10.3390/ijms27052314)

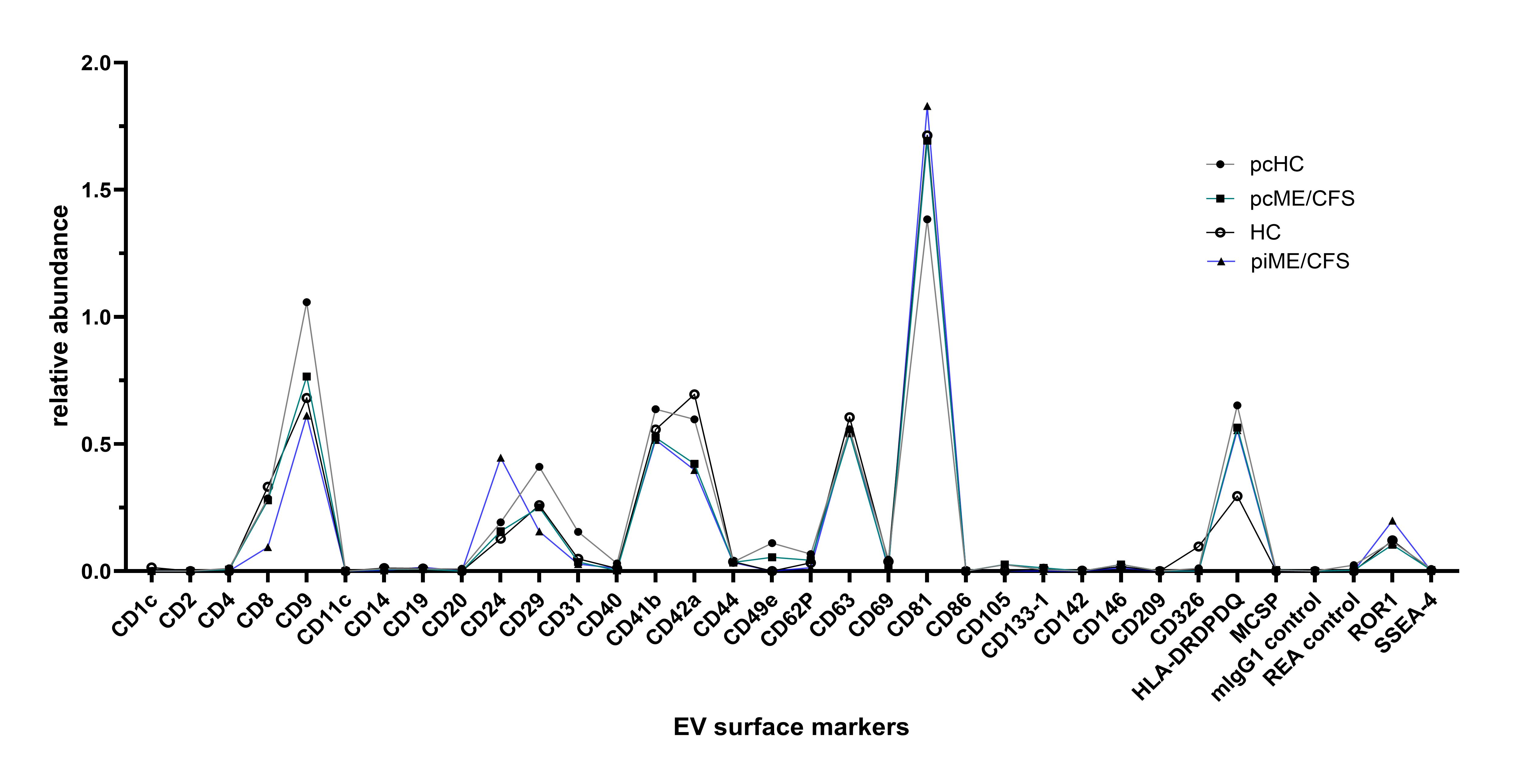

Supplement: Supplementary file 1 [file ijms-27-02314-s001.zip › Supplemental Figure_S1.tif]

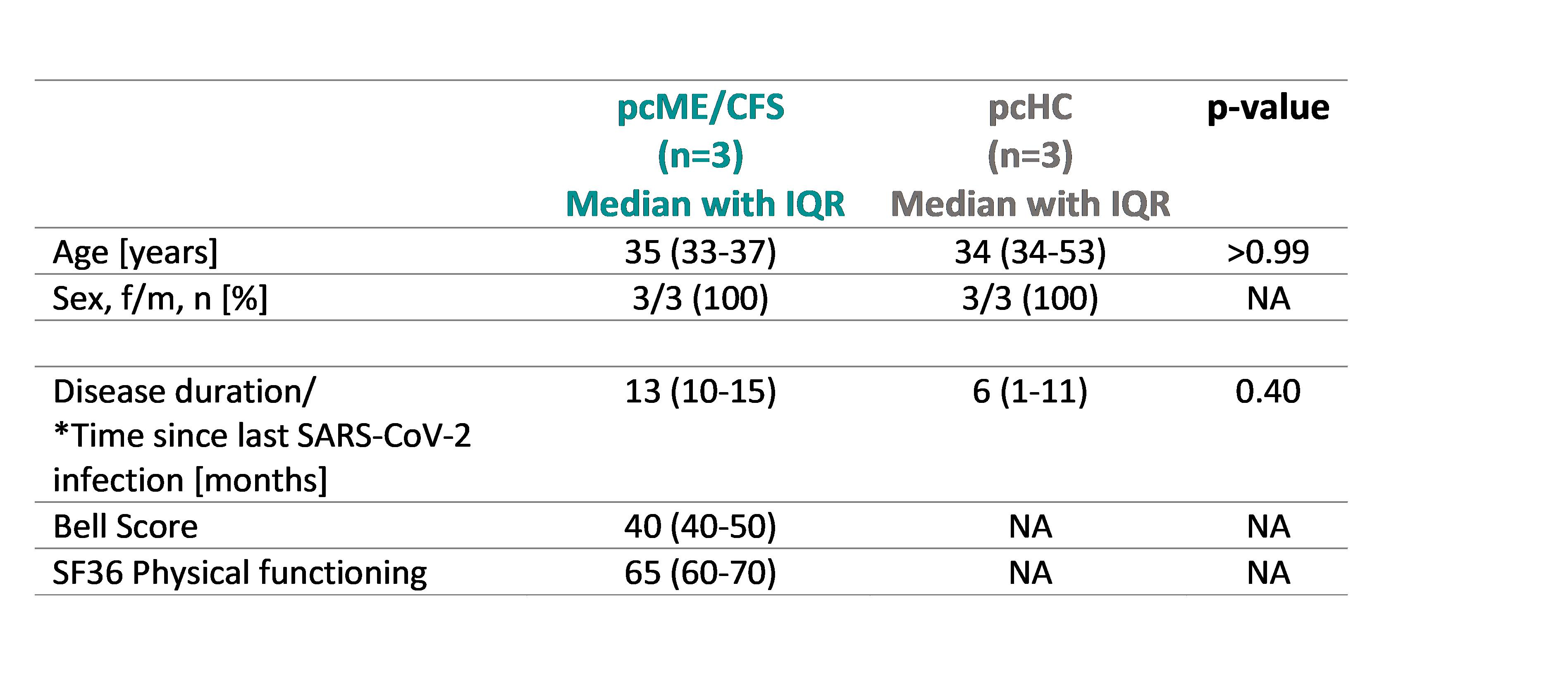

Supplement: Supplementary file 1 [file ijms-27-02314-s001.zip › Supplemental Table S1.tif]

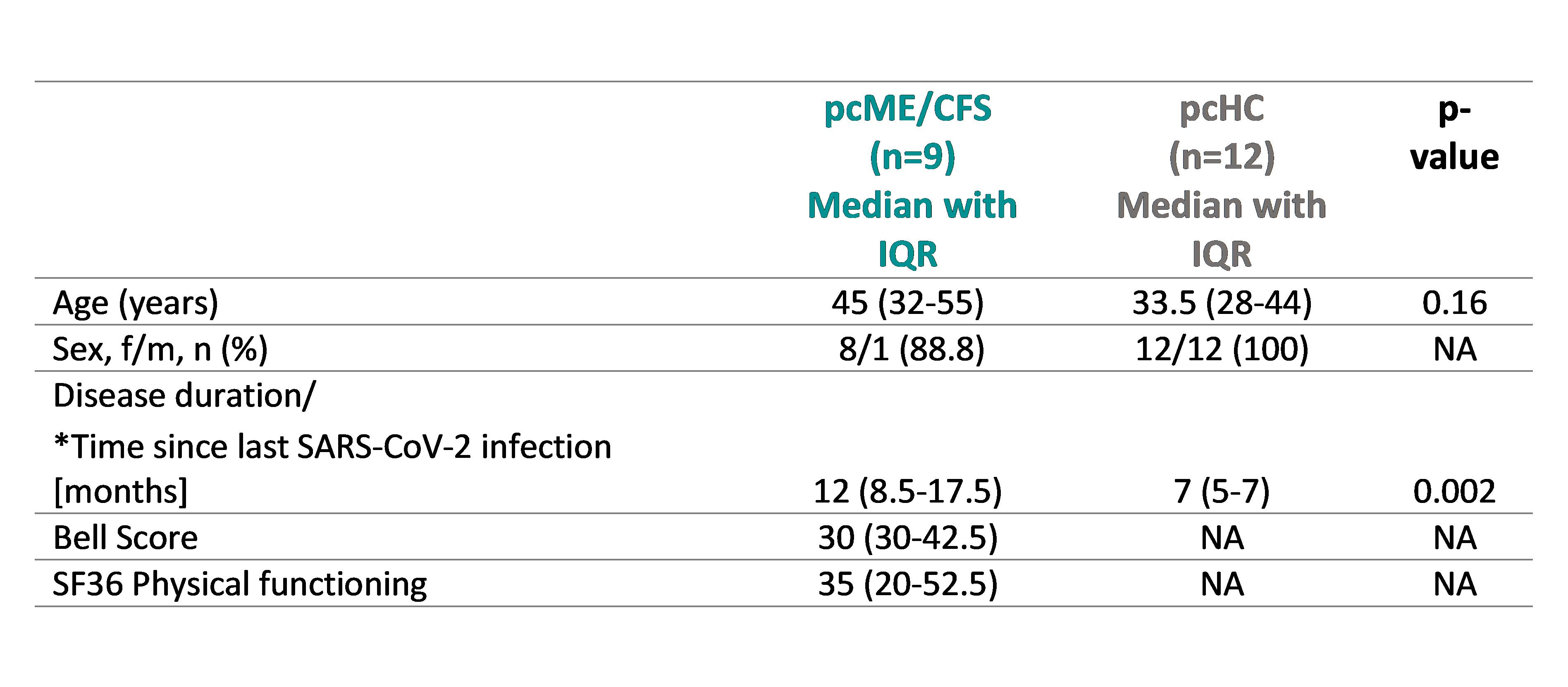

Supplement: Supplementary file 1 [file ijms-27-02314-s001.zip › Supplemental Table S2.tif]

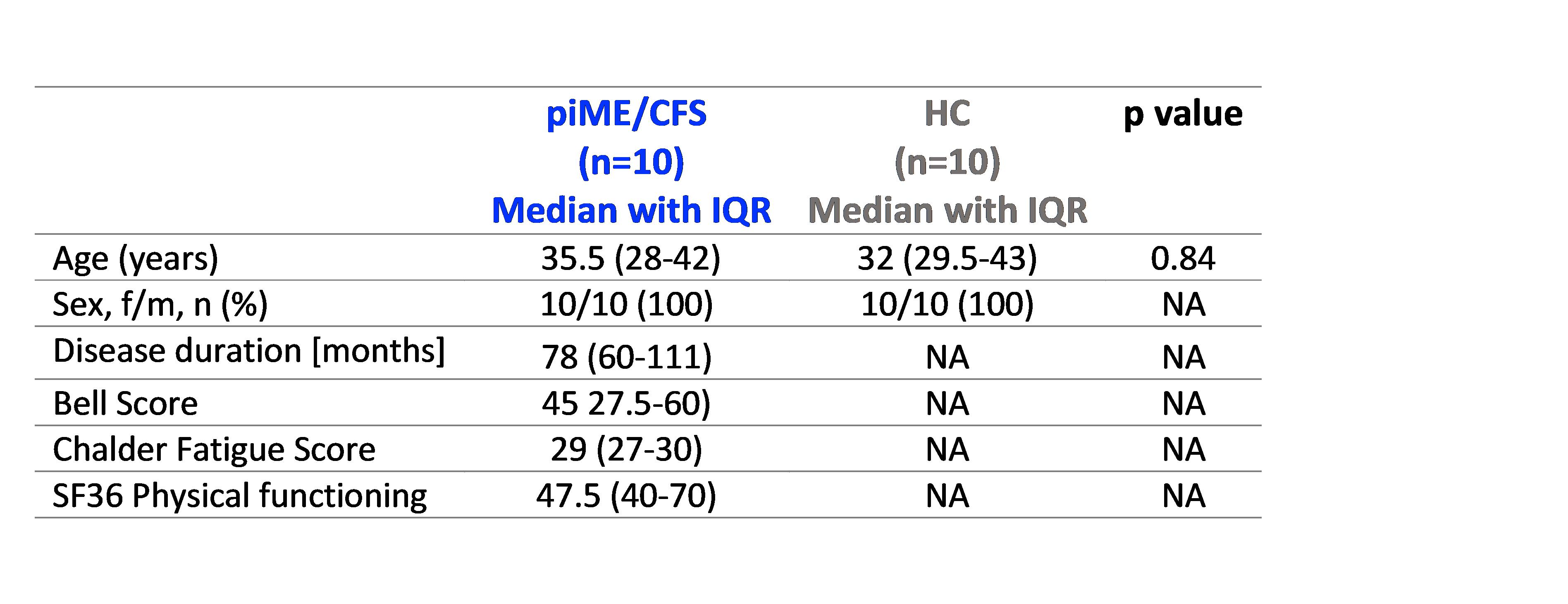

Supplement: Supplementary file 1 [file ijms-27-02314-s001.zip › Supplemental Table S3.tif]

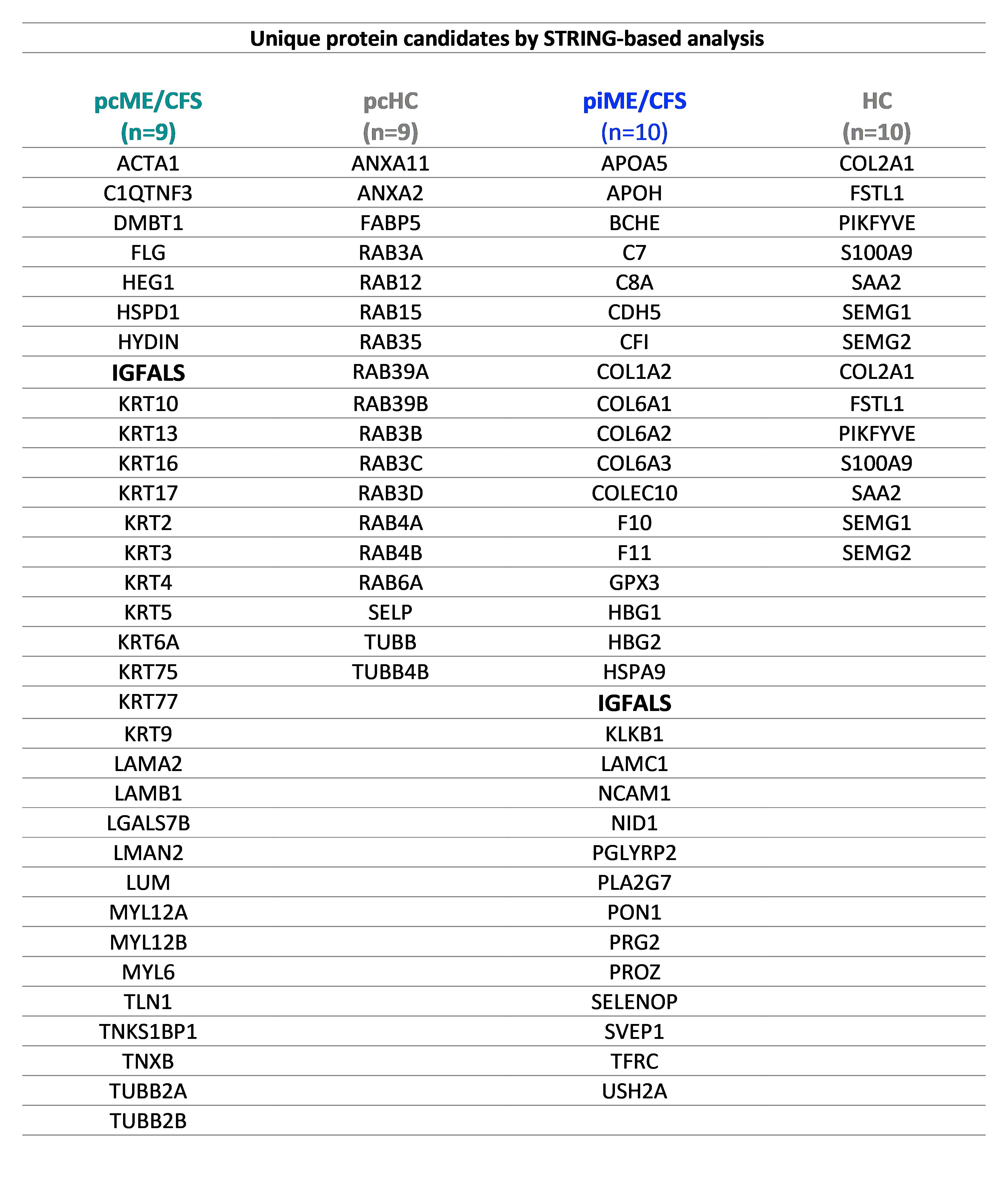

Supplement: Supplementary file 1 [file ijms-27-02314-s001.zip › Supplemental Table S4.tif]

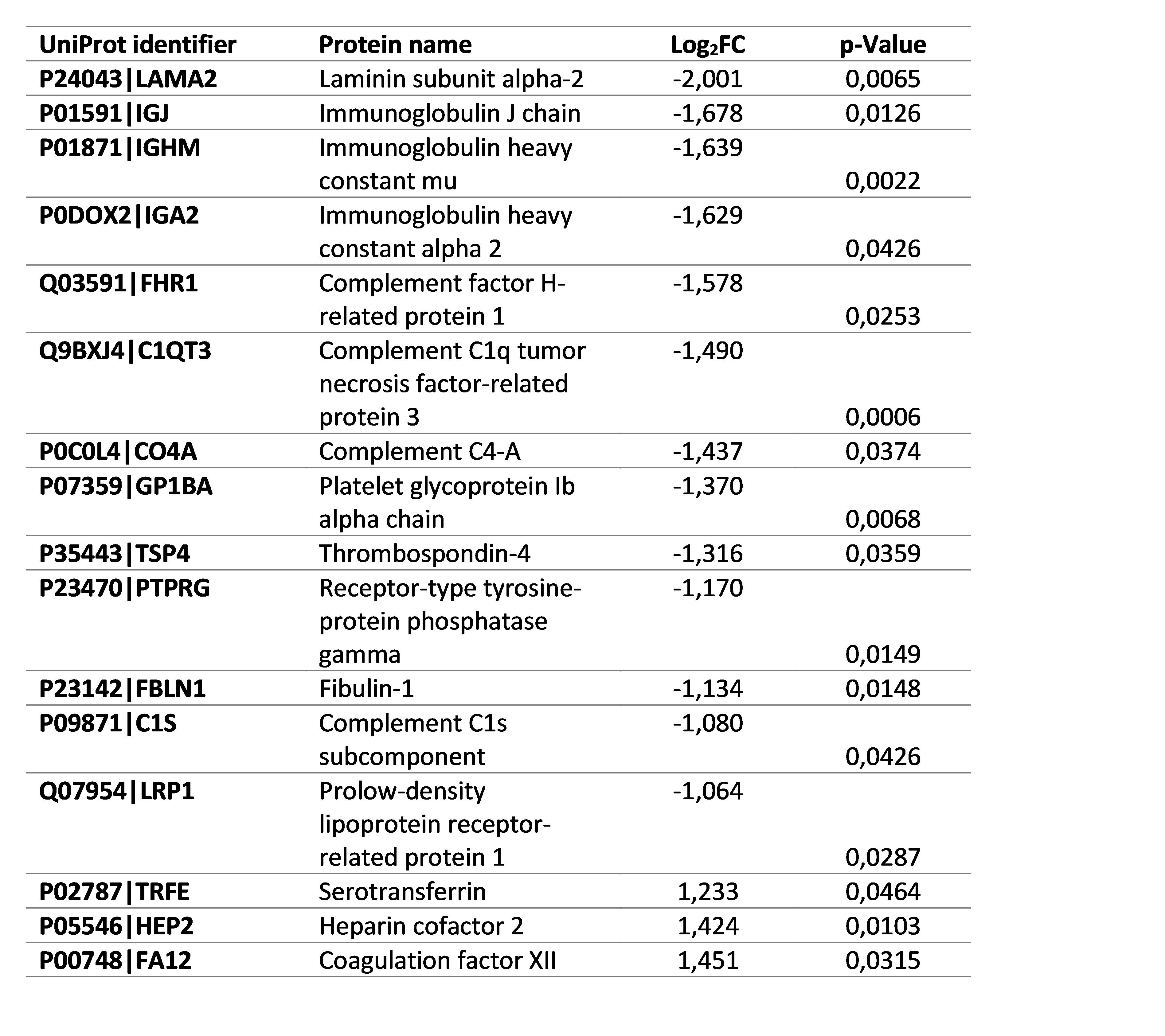

Supplement: Supplementary file 1 [file ijms-27-02314-s001.zip › Supplemental Table S5.tif]

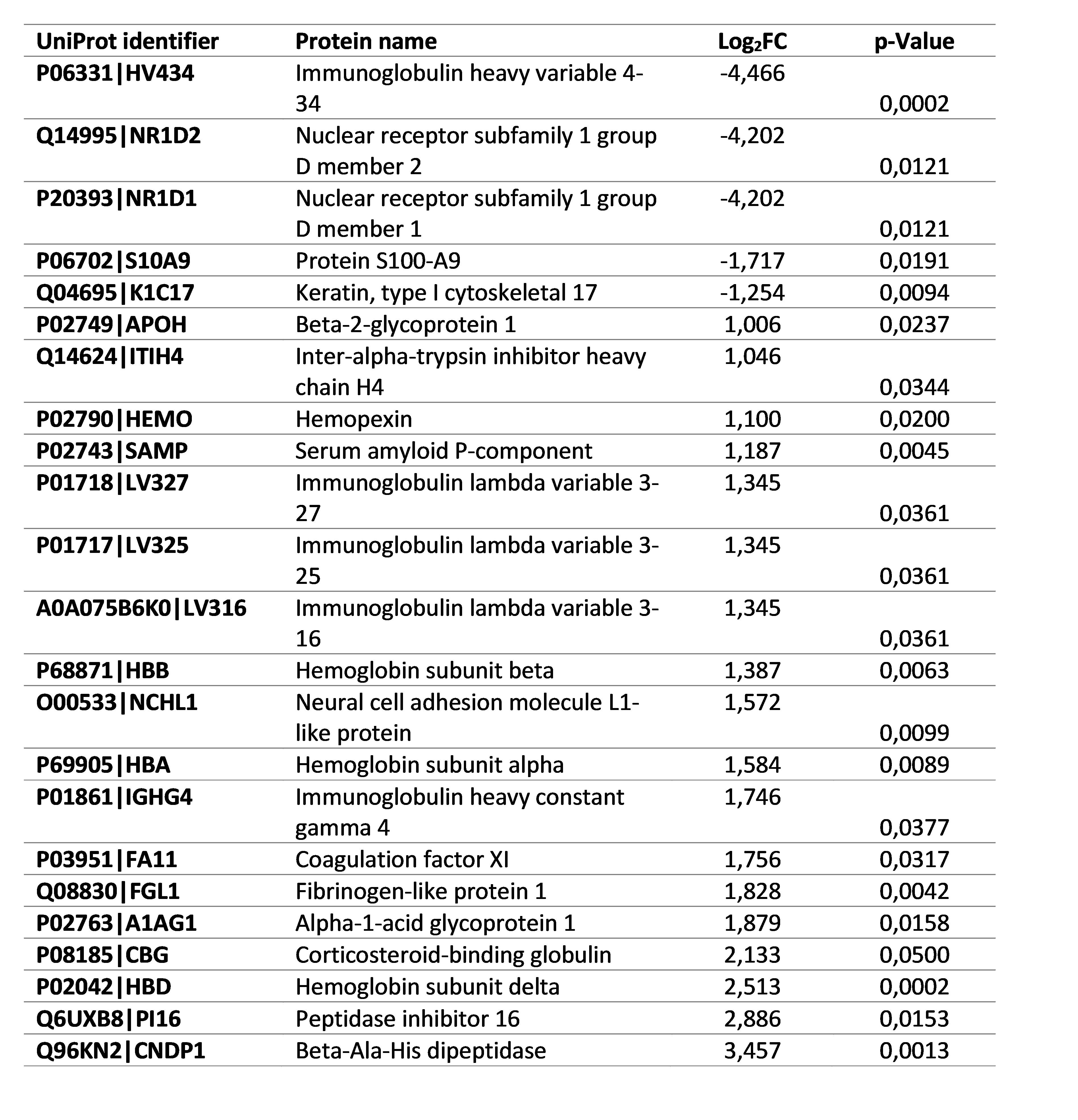

Supplement: Supplementary file 1 [file ijms-27-02314-s001.zip › Supplemental Table S6.tif]

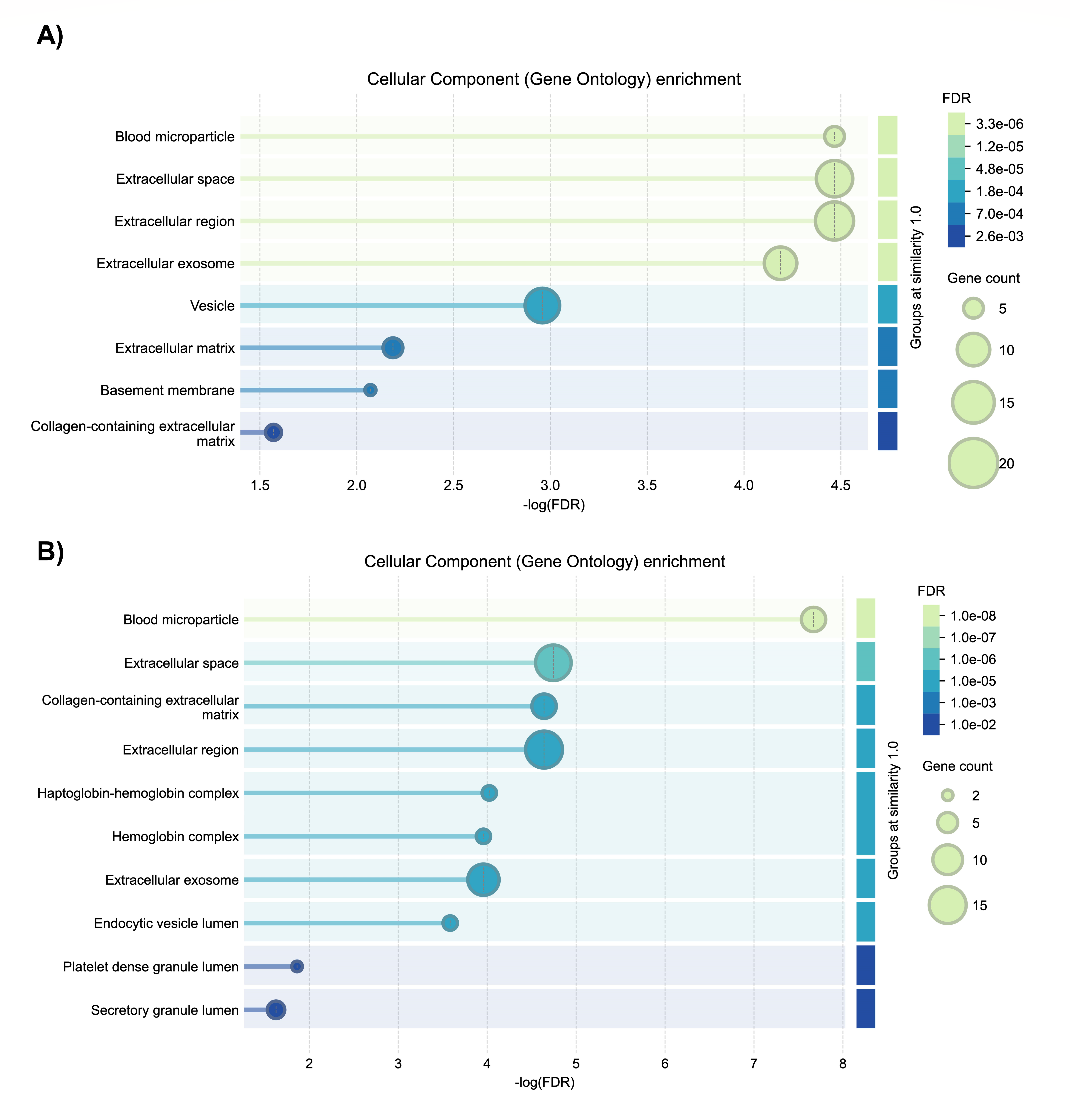

Supplement: Supplementary file 1 [file ijms-27-02314-s001.zip › Supplemental_Figure_S2.tiff]

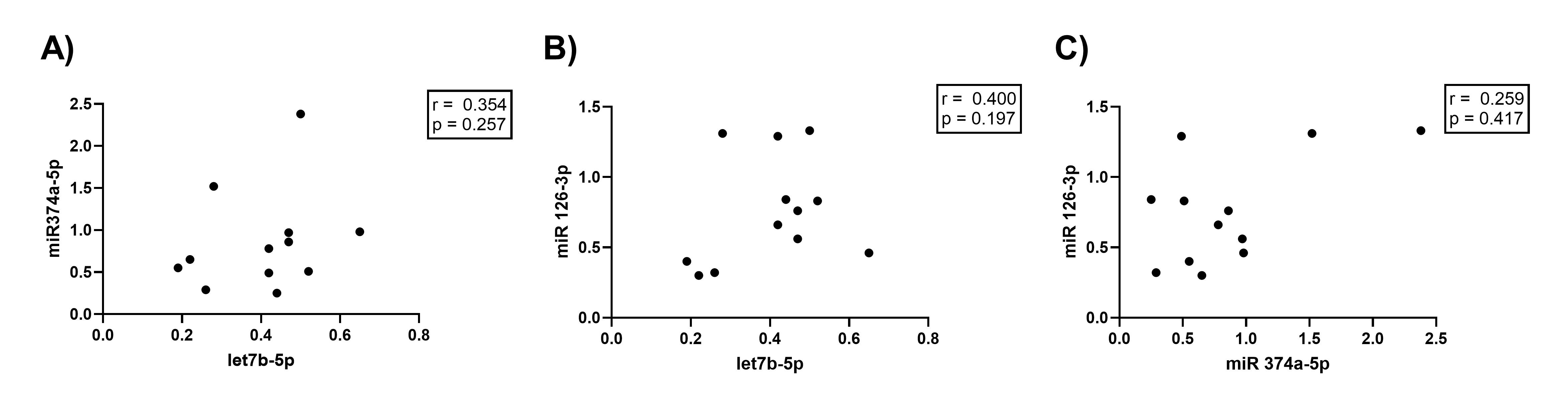

Supplement: Supplementary file 1 [file ijms-27-02314-s001.zip › Supplemental_Figure_S3.tif]
